# Nanoflow Size Exclusion Chromatography–Native Mass Spectrometry of Intact Proteoforms and Protein Complexes

**DOI:** 10.1021/acs.analchem.5c01019

**Published:** 2025-06-06

**Authors:** Ziran Zhai, Thomas Holmark, Annika A. M. van der Zon, Vasilis Tseliou, Francesco G. Mutti, Alina Astefanei, Andrea F. G. Gargano

**Affiliations:** † Analytical Chemistry Group, Van’t Hoff Institute for Molecular Sciences (HIMS), 1234 University of Amsterdam, Science Park 904, Amsterdam 1098 XH, The Netherlands; ‡ Centre for Analytical Sciences Amsterdam, Van’t Hoff Institute for Molecular Sciences (HIMS), University of Amsterdam, Science Park 904, Amsterdam 1098 XH, The Netherlands; § Biocatalysis Group, Van’t Hoff Institute for Molecular Sciences (HIMS), 1234 University of Amsterdam, Science Park 904, Amsterdam 1098 XH, The Netherlands

## Abstract

Native size-exclusion chromatography (SEC) coupled with native mass spectrometry (nMS) enables the characterization of proteins and protein complexes by combining liquid-phase separation (SEC) and mass measurement (nMS). This approach allows for an increase in the throughput of nMS experiments, reduces the bias that may be present due to the co-ionization of oligomers, and facilitates online sample buffer exchange. Conventional SEC-nMS uses volatile buffers and relatively wide-diameter columns (e.g., ≥1 mm), with flow rates in the tens of μL/min. To ionize sample components under this flow regime, relatively harsh electrospray ionization (ESI) desolvation conditions are needed, potentially resulting in protein dissociation/denaturation. Additionally, relatively large amounts of samples are required (several μgs). Herein, we describe the development of a nanoflow SEC-nMS method using 200 μm I.D. columns, operated at 500 nL min^–1^. This approach enables buffer exchange, oligomer separation, and mild ionization conditions (e.g., without the assistance of heated gas flow or temperature). Compared to microflow (1 mm I.D. column), the nanoflow method achieved a 4-fold increase in MS peak intensity, despite using a sample 20 times less concentrated (0.05 mg mL^–1^ for nanoflow vs 1 mg mL^–1^ for microflow). Furthermore, we evaluated the impact of three injection approaches on sensitivity and separation efficiency: large volume (1 μL), nanovolume (50 nL), and online mixed-bed ion-exchange capillary trap injection. To demonstrate its performance and applicability for sample-limited analysis, the final method using nanovolume injection was applied to several model proteins, protein complexes and a urine sample from a pregnant donor.

## Graphical abstract

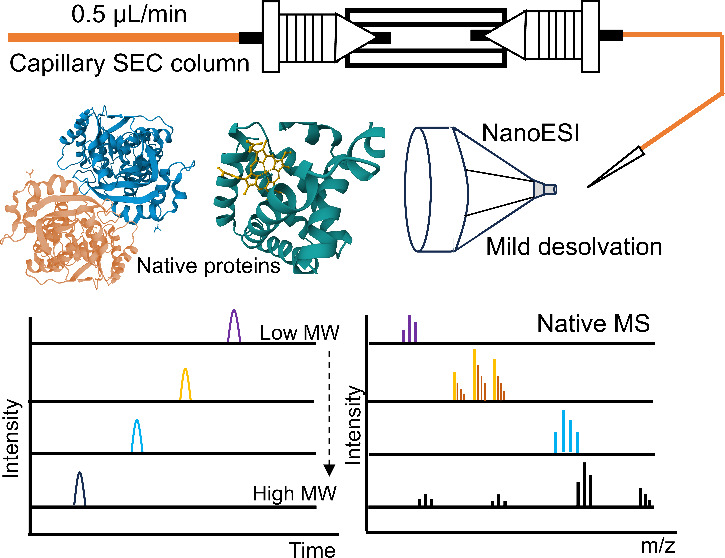

## Introduction

Native mass spectrometry (nMS) is increasingly applied in structural and molecular biology to analyze intact proteins, protein-small molecule interactions, and noncovalent complexes.^1–3^ With nMS, protein assemblies can be retained in the gas phase to get insights into their molecular composition and high-order structure.^4,5^ This is typically done using direct infusion approaches with static nanoelectrospray to infuse purified protein buffer exchanged into a solution with volatile salts at neutral pH.^1,6–8^ However, since no separation is present, this approach can only analyze samples with limited complexity, as dynamic range and signal suppression limit the analysis to the most abundant species in the solution.

Native separation methods can be coupled to native MS, allowing the handling of more complex samples. Examples are ion exchange chromatography (IEC),^9^ hydrophobic interaction chromatography (HIC),^10^ size exclusion chromatography (SEC),^11^ capillary electrophoresis (CE),^12^ and flow field flow fractionation.^13^ Among them, the SEC can separate various oligomeric states of protein complexes.^14,15^ In SEC, analytes are separated based on their hydrodynamic radius, which correlates to their size. The stationary phases are composed of porous silica particles chemically functionalized to avoid interaction with proteins. Larger proteins or oligomers access a lower fraction of the pore volume than smaller proteins.^16,17^ Native SEC methods have been applied to various applications, including native exosomes, plasma proteins, protein complexes, biopharmaceuticals, and aggregates.^18–20^ When establishing a method, critical parameters include the pore size of the stationary phase with respect to the radius of the protein, the chemistry of the stationary phase, and the composition of the mobile phases.^21^ To create nondenaturing analytical conditions and avoid secondary interactions (which can lead to effects such as protein adsorption, peak tailing, and band broadening), high concentrations of nonvolatile buffer systems (e.g., 200 mM NaCl in phosphate buffers at neutral pH) are used.^22,23^ However, nonvolatile buffers and salts are incompatible with MS measurements.^24^ Therefore, volatile additives such as ammonium acetate (AmAc) are employed to perform SEC-nMS.^14^

SEC is a nonretentive chromatographic approach and its separation performance can be severely affected by postcolumn dead volumes. As a result, the columns used in SEC-nMS typically have an internal diameter (ID) of 4.6 mm, with flow rates on the order of 100 μL min^–1^ or higher, and are either directly coupled to ESI-MS or coupled via a flow splitter.^20,25^ During ESI ionization under these conditions, heated gas flow in combination with high energy (e.g., electrospray voltage and in-source dissociation) is needed to perform thorough desolvation, which poses a threat of damaging labile or unstable proteins.^26–28^ Furthermore, relatively large sample quantities are needed (e.g., tens of micrograms), making these conditions unsuitable for sample-limited applications. To mitigate these challenges, low microliter flow SEC-nMS methods have been recently described. Hecht^29^ and Ventouri^11^ described a microflow SEC-nMS method (15 μL min^–1^) in a 1 mm ID column, reporting 10 to 100-fold gains in MS sensitivity and enabling the detection of labile proteins with softer ionization conditions, although heated gas (e.g., 80 °C) is still needed for solvent evaporation during ESI.

Nano-ESI is an effective approach to increase MS sensitivity and perform nMS experiments.^30,31^ Compared to conventional ESI, smaller droplets are initially produced, eliminating the need for heated auxiliary gases. The smaller droplets yield an increased surface charge-to-volume ratio that promotes more efficient ion formation and allows the methods to be more tolerant of buffer composition, detergents, and salt content.^32–35^ Despite the favorable MS ionization conditions, to our knowledge, native nanoSEC separation (<1 μL min^–1^) has not yet been coupled with nanoESI. Previous studies realized microscale SEC-UV separation as demonstrated by Rea et al.,^36,37^ where 300 μm ID columns were used at a flow rate of 1 μL min^–1^. Additionally, Smoluch et al. described peptide (up to 7 kDa) separations under denaturing conditions using SEC-MS at 2 μL min^–1^.^38^

In this study, we describe the development of a nanoflow size exclusion chromatography–native MS (nanoSEC-nMS) to characterize intact proteins and protein complexes (Scheme 1). Our strategy enables the direct coupling of capillary SEC (200 μm ID) columns to nMS at nanoliter flow rates (500 nL min^–1^) with mobile phases of up to 400 mM ammonium acetate (high buffer concentration compatibility), thereby avoiding extensive sample preprocessing and increasing analysis throughput. The reduced flow rate enabled higher-efficiency ionization without the use of heated gases, resulting in over four times higher detector sensitivity compared to our previously reported microflow analysis, even when analyzing a 20 times less concentrated sample.^11^ The mild desolvation also helps preserve the original structure of labile protein assemblies. Our method enables the identification of native protein assemblies across a wide molecular weight (MW) range (10 kDa to 240 kDa, reaching the limits of our MS instrument) and effectively separates their oligomeric states at the intact level. Importantly, the method’s sensitivity allows for the analysis of proteins from limited sample quantities (70 ng) within a real biological sample, as demonstrated by our analysis of urine from a pregnant donor.

**Scheme 1:**
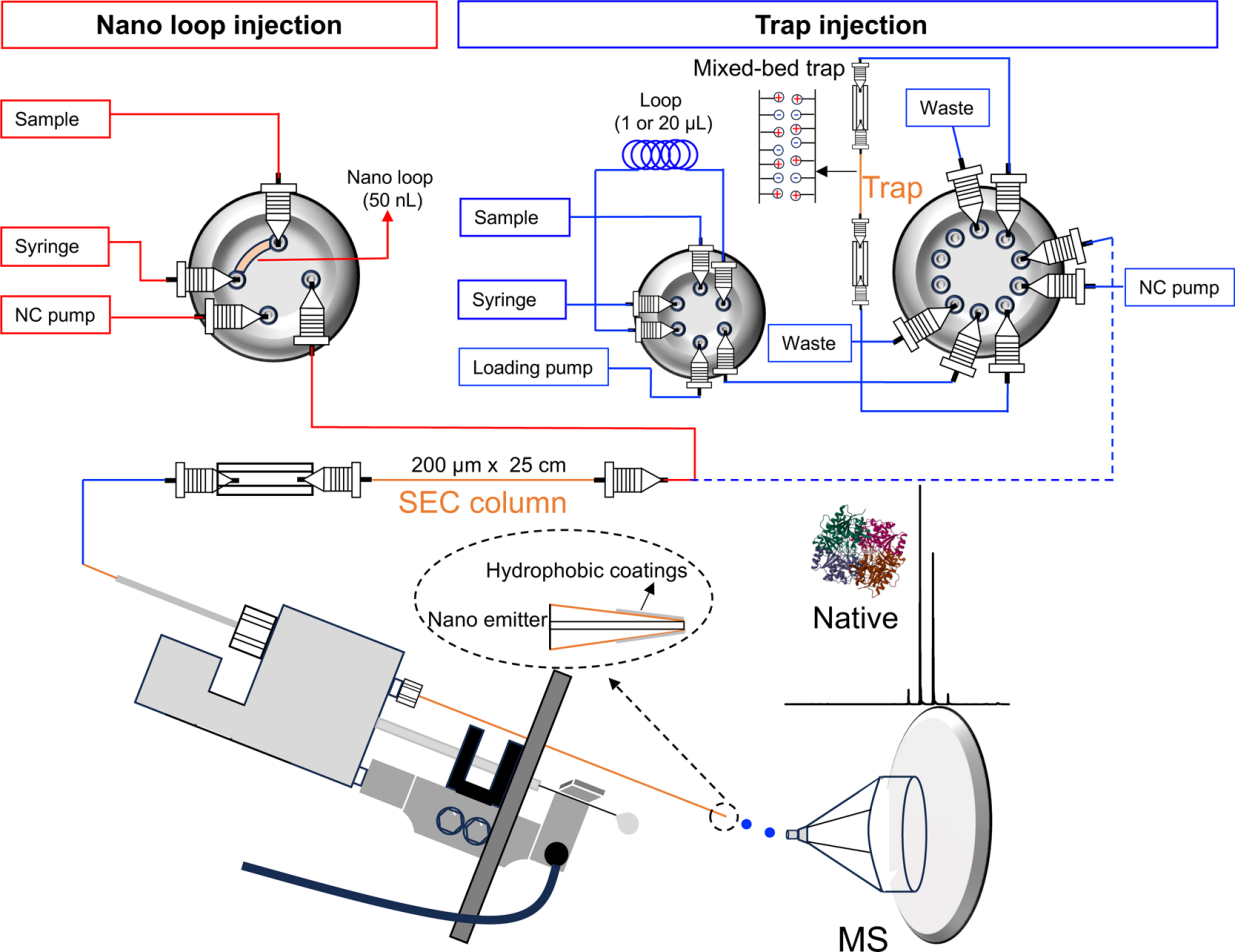
Illustration of the Nanoflow Size Exclusion Chromatography–Native Mass Spectrometry Setup

## Experimental Section

Details of the materials used are reported in section S1.1.

### Preparation of Capillary Columns

First, frits of approximately 4 mm length were made in empty fused silica capillaries (200 μm ID and 360 μm OD) by silica polymerization. The column was dipped in a mixture of potassium silicate and formamide. Frit polymerization was conducted in a column oven at 100 degrees for 12 h. SEC particles were obtained from a SEC column (7.8 × 300 mm × 5 μm × 250 Å, TSKgel G3000SWxl, TOSOH) and reconstituted in packing solutions with different compositions at a concentration of 300 mg mL^–1^ and ultrasonicated for at least 30 min to disperse uniformly. An illustration of the packing station is reported in Figure S1. The capillary SEC columns (200 μm ID × 250 mm) were fabricated by slurry packing the particles into 25 cm long capillaries with a Shimadzu pump (LC-10AD). Several packing solvents were tested: S1:20% MeOH, S2:200 mM AmAc, S3:50 mM PBS/50 mM NaCl, S4:50% ethylene glycol/2.5% Tween/47.5% water, S5: ACN, S6: IPA, and S7: MeOH. Cation and anion exchange resins (5 μm × 300 Å or 1000 Å) were employed to prepare the mixed-bed ion exchange trap capillary columns (200 μm ID × 4 cm). Stationary phases were mixed with a ratio of 1:1 to a total concentration of 25 mg mL^–1^ and packed following the same procedure described for SEC columns, using 50 mM PBS (pH 7.0) as the buffer. All capillary columns were kept under pressure for at least 10 min after the desired length was packed to ensure good packing homogeneity.

### NanoLC-UV Evaluation of Capillary SEC Columns

The performance of capillary SEC and mixed-bed trap columns were tested on an UltiMate RSLCnano system (Thermo Fisher Scientific, Breda, The Netherlands) equipped with a high-pressure pump with microflow selector, a loading pump (NCS-3500RS), a binary nano/capillary pump (NCP-3200RS), a column compartment (equipped with two ten-port, two-position valves), an autosampler (50 nL, 1 and 20 μL loops) and a UV detector (VWD-3400RS) with 3 nL flow cell. A detailed description of the injection setup is reported in 2.3.

The autosampler was connected to the inlet of the column, which was then connected to the UV cell. Twenty μm ID empty capillaries were used to make the connections. Uracil was used as a dead-volume marker compound to evaluate the packing efficiency with a concentration of 0.1 mg mL^–1^ (diluted by 20 mM PBS). 0.2 μL was injected into the column at a 0.5 μL min^–1^ flow rate and eluted with 200 mM PBS (pH 6.8). Additionally, the impact of filters (no filter, plastic filter, metal filter, and silica frits) in the flow path on chromatographic performance was evaluated. 50 nL of uracil was injected at a concentration of 2 mg mL^–1^ without the capillary SEC column and eluted with 200 mM PBS (pH 6.8) at a flow rate of 0.5 μL min^–1^. During the analysis, the monitored UV wavelengths were set at 214, 256, and 280 nm.

### Micro and nanoSEC-nMS

A Q-Exactive Plus Biopharma (Thermo Fisher Scientific, Bremen, Germany) was used in this study. For the nanoflow SEC-nMS method (Figure S2), the MS was operated in positive mode, with a spray voltage of 1.8 kV, a transfer capillary temperature of 275 °C, and an S-lens of RF 200. The nanosource was made of a nanospray-flex ESI (Thermo Fisher Scientific) and a Simple Link UNO (1/32, Fossiliontech, Albacete, Spain). Three different ESI nanoemitters were tested: PicoTip (10 μm I.D. × 105 mm, silica, FS360-20-10-N-5-105CT, New Objective), EASY-Spray (30 μm I.D. × 40 mm, metal, ES542, Thermo), and Lotus (20 μm I.D. × 75 mm, hydrophobic coating, ref.20-7.5, Fossiliontech).

The MS acquisition parameters were: HRM mode; scan range, 400 to 8000 *m*/*z*; in-source collision-induced dissociation (isCID) values vary for different proteins (see the figure captions); number of microscans, 10; resolution, 17,500; automatic-gain-control (AGC) target, 3 × 10^6^; maximum injection time (IT), 200 ms. The microflow method used the same MS acquisition settings but different ionization conditions. For details of the method and part used, we refer to the work from Iro et al.^11^

The nanovolume injection was performed using a four-port, two-position valve (Cheminert C74H-1674-.05, VICI). 1 mg mL^–1^ of proteins dissolved in 400 mM AmAc were analyzed at a flow rate of 0.5 μL min^–1^ using packed capillary SEC columns by elution solvents of 200 or 400 mM AmAc with 50 nL injection. The large-volume injection was performed using a fixed loop of 1 μL. 0.05 mg mL^–1^ of proteins dissolved in 400 mM AmAc were analyzed at a flow rate of 0.5 μL min^–1^. Details of the trap-elute injection method are shown in Supporting Information S1.2 and S1.3. The internal diameters of the connecting tubes (silica capillaries) are below 50 μm.

### Urine hCG and Ovitrelle Protein Analysis

Urine from a donor in the tenth week of pregnancy was collected and used to identify human chorionic gonadotropin (hCG) proteins. Approximately 10 mL of the urine sample was dialyzed overnight against 50 mM potassium phosphate buffer (pH 7.5) and subsequently concentrated to 700 μL using a Vivaspin 20 concentrator (30,000 MWCO). The Bradford assay and SDS-PAGE were used to roughly determine the concentration of urine samples and check the protein distributions. Recombinant hCG (Ovitrelle) served as the reference protein. HILIC-MS was performed following the reported procedures to confirm the structures of hCG and Ovitrelle (see Supporting Information, Section S1.4 to S1.6, for details).^39^

### Data Analysis

The LC-UV data were analyzed using Chromeleon 7.2.10 (Thermo, USA). The MS data were analyzed by Freestyle software (version 1.7, Thermo Fisher Scientific). UniDec v7.0.2 software^40^ (University of Arizona, Tucson, AZ, USA) was employed for the deconvolution of mass spectra. The deconvolution parameters are reported in Table S1. Total ion chromatogram (TIC) and extracted ion chromatogram (EIC) were smoothed using a 7-point Gaussian filter. Formulas used to evaluate results are shown in Table S2. The data presented are available at the following zenodo repository link: https://zenodo.org/records/15596123.

## Results and Discussion

Native size exclusion chromatography (SEC), while a nonretentive separation technique with limited peak capacity (typically around 10–20), is a valuable method for preserving protein assemblies and partially resolving oligomeric states in samples of moderate complexity. Direct coupling of SEC to MS is usually performed using columns with internal diameters above 1 mm, flow rates in the range of tens of μL min^–1^, and salt-rich separation conditions. The high flow rates applied require harsh ionization conditions, restricting the application of SEC-MS to the analysis of proteins and protein complexes available in relatively large quantities and that can withstand higher energy desolvation (e.g., mAbs).

Herein, we describe a nanoSEC-native MS (nMS) method for the analysis of noncovalent complexes. This method offers key advantages over current microflow SEC-nMS approaches, reducing sample consumption and providing gentler ionization conditions to preserve noncovalent interactions, thus broadening the scope of SEC-nMS for biological investigations. To achieve nanoSEC-nMS for noncovalent complex analysis, we (i) optimized key parameters, including column packing (frits, solvent slurry packing composition), emitter selection, and injection strategy to ensure efficient separation and performance. (ii) The method was compared to state-of-the-art microflow SEC-MS. (iii) Finally, its applicability was demonstrated through the analysis of reference protein complexes and a urine sample from a pregnant donor.

### Packing and Performance Evaluation of Capillary SEC

Capillary SEC columns were packed in our laboratory using packing materials obtained from commercially available columns. Many factors can affect the quality of packed capillary columns, such as packing solvents, pressure, slurry density, column length, bed heterogeneity, etc.^41–43^ In our study, we investigated the influence of packing solvents on the efficiency of the nanoSEC columns. Prior to testing the performance of slurry-packing conditions, we tested the influence of different frits/screens used to contain the particles in the capillary column. We compared the metal screen, the plastic screen, the silica frits, and the absence of frits/screen in the analysis of uracil (Figure 1a). In the case of the silica frits, extra dead volumes were present to make a connection; a capillary of about 3 cm × 0.2 mm ID had to be used to realize the connection with the UV detector. Using the metal and plastic screen, peak-width at half height (*w*_h_, min) was 0.55 and 0.65, respectively, which is higher than that of silica frits (0.40) and when no screen (0.30) is used. In addition, the metal screen causes peak tailing, which can be measured by the increase in the peak asymmetry from 1.70 (no screen) to 7.55. This value is significantly higher than that of plastic screens (3.42) and silica frits (2.43). Based on these results, we selected silica frits as the optimal way to restrict stationary-phase resins inside capillary columns because of the lower peak tailing (asymmetry: 2.43) and higher chromatographic performance (*w*_h_: 0.40).

**Figure 1:**
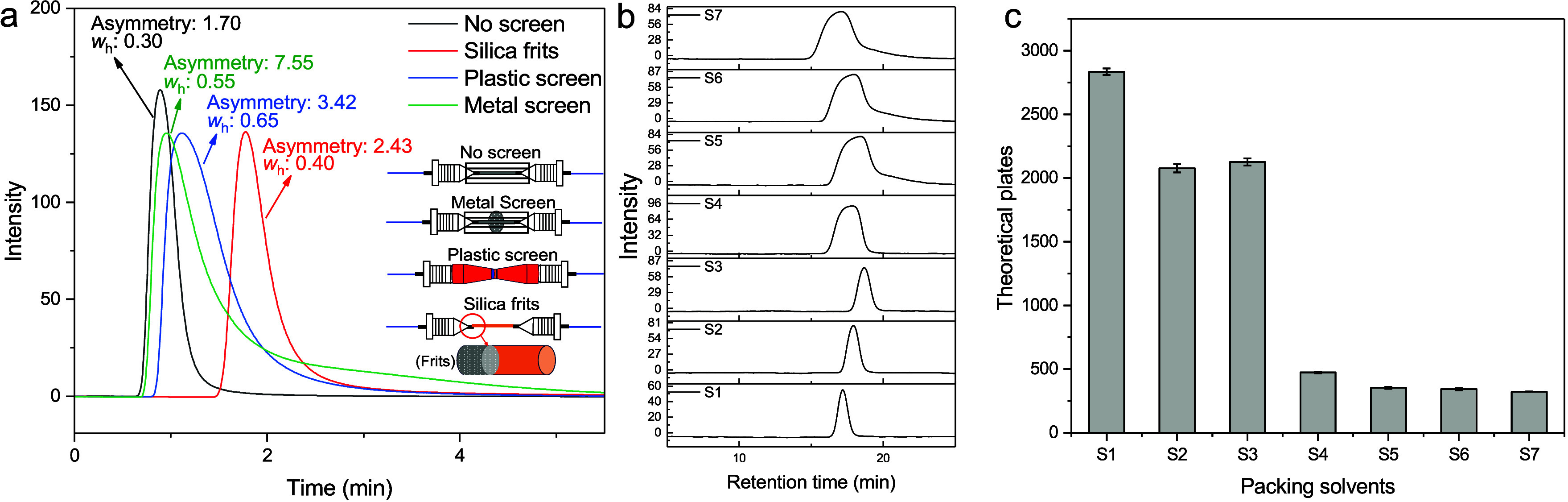
(a) UV chromatograms obtained from the injection of uracil without columns to evaluate the influence of different types of screens/frits (no screen/frits, metal screen, plastic screen, and silica frits) on the peak shape and dispersion (the capillaries are empty and do not contain the stationary phases). It should be noted that when testing the silica frit, a larger volume had to be used to realize the connections between the tubing, leading to a delay in elution and further dispersion. (b) Performance evaluation of SEC capillary columns prepared with various packing solvents (S1 to S7) using SEC-UV analysis of uracil. (c) Theoretical plates of capillary columns in (b). S1 to S7 were 20% MeOH, 200 mM AmAc, 50 mM PBS/50 mM NaCl, 50% ethylene glycol/2.5% Tween/47.5% water, ACN, IPA, and MeOH.

Next, we tested the effect of different solvent compositions on the slurry suspension employed for packing columns. When packing capillary columns with slurry, the stationary-phase materials should disperse uniformly in the solvents and remain suspended throughout the packing time.^44^ To assess this, we tested seven solvents previously reported in the literature, making slurries with the same particle concentration: S1:20% MeOH, S2:200 mM AmAc, S3:50 mM PBS/50 mM NaCl, S4:50% ethylene glycol/2.5% Tween/47.5% water, S5: ACN, S6: IPA, and S7: MeOH.^44^ Particle suspensions were visually inspected after 0.5 and 2.5 h (pictures are collected in Figure S3). Of the solvents tested, the slurry remained dispersed longer under conditions S1 and S4, exhibiting less precipitation. Thereafter, we packed capillary SEC columns using silica frits and various packing solvents S1–7 described above. Uracil was used to evaluate the performance of homemade capillary SEC columns. As shown in Figures 1b and 1c, 20% MeOH was the solvent that gave the best performances in terms of theoretical plates (above 2,750) and peak asymmetry (1.06; the complete results are summarized in Table S3) and was therefore selected as the packing solvent.

### Selection of Emitters for Nanoflow SEC-nMS

As a starting point for the development of our nanoSEC-nMS method, we selected mobile phase conditions that we previously discussed for microflow SEC-nMS.^11^ In particular, we used 200 mM ammonium acetate at a pH of 6.8 and a flow rate of 500 nL min^–1^. The high salt concentrations and the absence of desolvation gases during nanoelectrospray make the coupling to MS susceptible to spray instability and emitter clogging. Therefore, we tested the nanoESI stability of three emitters: metal, uncoated silica, and silica emitters coated with a hydrophobic polymer. Metal and uncoated emitters were easily clogged after around 15 min of the electrospray. The clogging was visually observed at the tip of the emitter due to the salt precipitation and resulted in a significant pressure increase (over 100 bar). We suggest that with these emitters, liquid accumulates at the tip, and the hydrophilicity of the outer emitter surface increases the area where the ESI meniscus forms. During evaporation, residue accumulates, which leads to emitter clogging (a schematic illustration is reported in Figure S4). When the emitter with hydrophobic coatings was applied, the liquid did not wet the tip, forming a round nanodroplet even without the voltage. This allows the liquid to evaporate with minimal interaction with the outer surface of the emitter. Using these hydrophobically coated emitters, we could perform nanoSEC-MS with the same emitter for up to weeks. We tested elution solvents with concentrations up to 400 mM ammonium acetate without significant problems for the emitter and obtained good protein signals.

### Nanoflow SEC-nMS Performance: Comparison of Injection Approaches

Next, we investigated the sensitivity of nanoSEC-nMS with respect to the microflow method we recently described.^11^ For microflow experiments, a HESI (using a microflow emitter) source allowed to use of gas and a high-temperature environment during ionization. The two methods were compared by injecting the same volume of a BSA solution (1 μL), and similar linear flow velocities were applied (about 0.2 mm s^–1^). Preliminary results showed that the nanoSEC-nMS response was significantly higher. Therefore, we reduced the sample concentration injected in the nanoflow method from 1 mg mL^–1^ (used in microflow analysis) to 0.05 mg mL^–1^. Figure 2a compares the two methods. Despite the difference in concentration (20 times lower), the intensity of the extracted ion chromatogram (EIC) was around 4 times higher (3.0E6 vs 1.2E7). This is due to the combined effects of the reduced elution volume of the protein during nanoSEC (nanoflow peak volume based on peak width is 4.2 μL vs microflow 54 μL), higher column volume load (nanoflow 21.2% vs microflow 0.7% of a column volume), and the increased desolvation efficiency in nanoESI.^45^

**Figure 2:**
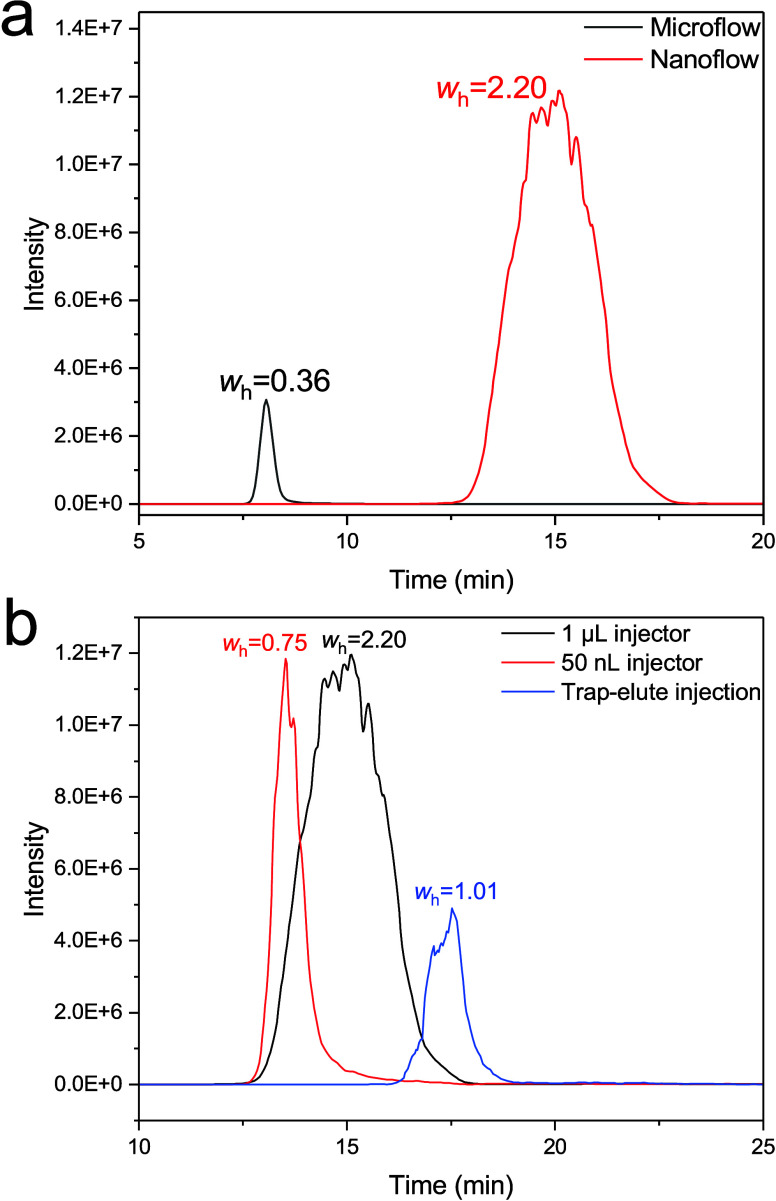
(a) Extracted ion chromatogram (EIC) of BSA measured by microflow and nanoflow SEC-nMS. The black line represents 1.00 mg mL^–1^ of BSA analyzed by microflow SEC-nMS (15 μL min^–1^). The red line represents 0.05 mg mL^–1^ of BSA analyzed by nanoflow SEC-nMS (0.5 μL min^–1^). The injection volume for both is 1 μL. (b) EIC of BSA measured by nanoflow SEC-nMS with different injection approaches: 1 μL injector, 50 nL injector, and trap-elute injection. The black line (1 μL injector) was obtained from a 1 μL injection of 0.05 mg mL^–1^ of BSA. The red line (50 nL injector) was obtained from a 50 nL injection of 1 mg mL^–1^ of BSA. The blue line (trap-elute injection) was obtained from a 1 μL injection of 0.05 mg mL^–1^ of BSA. The *m*/*z* values used for EIC in (a) and (b) can be found in Table S5.

However, the separation performance under nanoSEC conditions described here is significantly compromised (microflow: 3523 N vs nanoflow: 256 N). One of the significant sources of band broadening arises from the large volume injection. While this makes the setup suitable for buffer exchange purposes, it limits the ability to separate, for instance, oligomeric states of proteins. Therefore, to improve the separation performance of the method, we investigated the effect of different injection approaches.

Three different approaches were tested: 1 μL injector, a nanovolume injection valve with a fixed loop volume of 50 nL (corresponding to approximately 1% of the column volume), and a trap-elute setup/online SPE approach using a mixed-bed trap column composed of strong cation and anion exchange resins. Details of the development of the trap and elute method using different types of stationary phase selectors (weak and strong exchangers) and pore sizes (300 Å and 1000 Å) are reported in the Supporting Information (S1.2 and S1.3). We compared the three injection methods using BSA as a model analyte. During the analysis, the same amount of analytes was injected into the column (50 ng of BSA; 0.7 pmol). Results for the analysis of BSA are shown in Figure 2b. The *w*_h_ (min) of the 1 μL injector was around three times higher than the 50 nL (2.20 vs 0.75 min; corresponding to 256 and 1800 theoretical plates). The trap-elute injection method had in-between results with a *w*_h_ of 1.01 min (1667 theoretical plates). The intensity of BSA measured with the trap-elute approach was lower than the other two methods (4.0E6 vs 1.2E7), presumably due to sample loss in the trapping period or incomplete elution. To increase recovery, the elution with both 200 and 400 mM AmAc was tested (Figure S5), resulting in an increase in intensity (2.5E6 vs 1.5E7). Finally, MS analysis (Figure S6) revealed that the most abundant charge state with the 1 μL injector and trap-elute injection (+16) is higher than the 50 nL injector (+15). This was also confirmed when analyzing other proteins, such as enolase (Figure S7).

Although the trap-elute setup shows promise in increasing the volumetric load on the column (about 5 μL could be injected, exceeding 100% of the nanoSEC column volume), and thus enhancing detector sensitivity, the application of this approach revealed that the protein recovery may be incomplete (recovery of about 59.18%, Table S4). Additionally, the interaction between the protein and the stationary phase may alter the protein structure. Furthermore, the trap-elute method does not apply to all proteins; those with pI around 7 may not be captured by the trap columns. In contrast, the nanoinjection required relatively higher concentrations but gave the best chromatographic separation performance results. Therefore, we decided to adopt this injector for our nanoSEC-MS experiments.

In summary, while optimizing nanoSEC column conditions and sample injection significantly enhanced chromatographic performance, the resulting resolution was lower than microflow (e.g., BSA dimer/monomer resolution of 0.9 vs 1.3). Nevertheless, the substantial sensitivity gains of nanoSEC-nMS, coupled with nMS selectivity, provide a compelling analytical strategy for sample-limited biological analyses.

### Nanoflow SEC-nMS Analysis of Reference Proteins and Protein Complexes

To assess the performance of our method, we investigated a series of proteins and complexes with different molecular weights (MW), heterogeneity (e.g., post-translational modifications), and oligomeric states. Our setup allowed us to measure proteins without buffer exchange (typically a prerequisite in native MS experiments, also of reference proteins),^46^ as the nanoSEC separation allows to separate the salts from the protein elution area. In Figures 3a, 3b, and Figure S8, we report the results from proteinase K (PK), ovalbumin (Oval), alcohol dehydrogenase (ADH), and catalase (Cata). The corresponding EICs are reported in Figure S9. In all cases, reduced charge states (number and distribution of charges) typical of native MS experiments were observed. Moreover, most proteins did not present significant salt adducts. In the analysis of PK (protein with an MW of 29 kDa), we observed limited Na^+^ adducts and detected the protein in the *m*/*z* area of 2500–3500 distributed over 3 charge states (+8 to +10, Figure 3a). When analyzing ovalbumin (45 kDa), several glycoforms were observed, divided over 4 main charge states (+10 to +13) centered in the 3500 to 4500 *m*/*z* range (Figure 3b). ADH (147 kDa) was detected as a protein complex existing primarily as a tetramer with four charge states (+23 to +26) located between 5500 and 6500 *m*/*z* areas (Figure S8a). Finally, catalase (Cata), a protein composed of four subunits with an MW exceeding 240 kDa, was maintained as a tetramer, covering 6500 to 8000 *m*/*z* areas and reaching the maximum *m*/*z* of our MS instrument (Figure S8b). The low abundance and broad *m*/*z* signal observed from Cata reflect transmission issues of our instrument at high *m*/*z* and incomplete desolvation.

**Figure 3:**
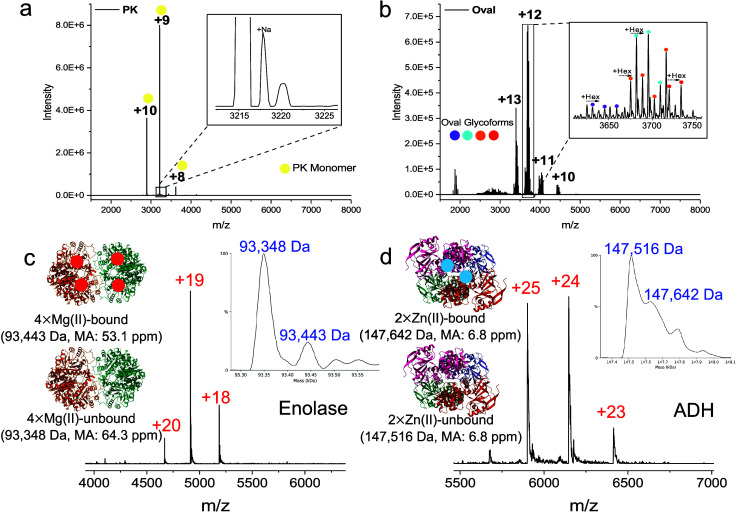
(a, b) Mass spectra of various proteins and complexes measured by the nanoflow SEC-nMS. (a) Proteinase K (PK); (b) ovalbumin (Oval). 50 nL of these proteins (1 mg mL^–1^) was injected into the system and eluted with 200 mM AmAc. Values of isCID for these proteins (a to b) are 55 and 35 eV, respectively. (c, d) Analysis of proteins coordinated with metal ions by the nanoflow SEC-nMS: (c) enolase; (d) alcohol dehydrogenase (ADH). 50 nL of both proteins (1 mg mL^–1^) was injected into the system and eluted with 200 mM AmAc. The values of isCID for both proteins are 15 eV. The mass of each protein was obtained with the deconvolution methods shown in Table S1. MA: mass accuracy.

Next, we checked the capability of the nanoSEC-nMS to retain small molecules and metal ions. These binding partners can be critical to protein folding and their activity.^47–49^ In particular, analyzing proteins coordinating with metal ions requires milder conditions to maintain the noncovalent interactions. Notably, the interface parameters, such as the source temperature, HCD pressure, and voltages, have a significant influence on their detection.^50^ Interestingly, using our nanoSEC-nMS method, we observed complexes with small molecules and metal ions. For example, two forms of Myo (with and no heme group) could be observed with MWs of 17,565 Da and 16,950 Da (Figures S10 and S11a).^51,52^ ConA was observed with mass differences that indicate possible binding to Mn (II) and Ca (II) (Figure S11b).^52^ Also, in the case of enolase, mass differences indicating the binding of two magnesium ions per subunit were observed (Figure 3c, 93,443 Da with Mg vs 93,348 Da without Mg).^46,53^ This was also the case for ADH (Figure 3d), where two Zn (II) cofactors were tentatively assigned (147,642 Da) compared to the ion-unbound state (147,516 Da).^46^ Table S6 lists the mass accuracy of assignments between the theoretical and observed MWs.

In addition, we tested the performance of our nanoSEC method in resolving oligomeric complexes by applying it to the analysis of l-asparaginase. This biopharmaceutical consists of several oligomers. Our method can preserve native states of the intact proteins and protein complexes while offering a partial liquid-phase resolution of different oligomeric species present in a sample. We were able to observe and separate the monomer, dimer, tetramer, and octamer forms of l-asparaginase (Figures 4a and 4b). Similarly, we were able to partially separate oligomers of enolase and BSA (results presented in Figure S12), demonstrating that nanoSEC can effectively separate oligomers.

**Figure 4:**
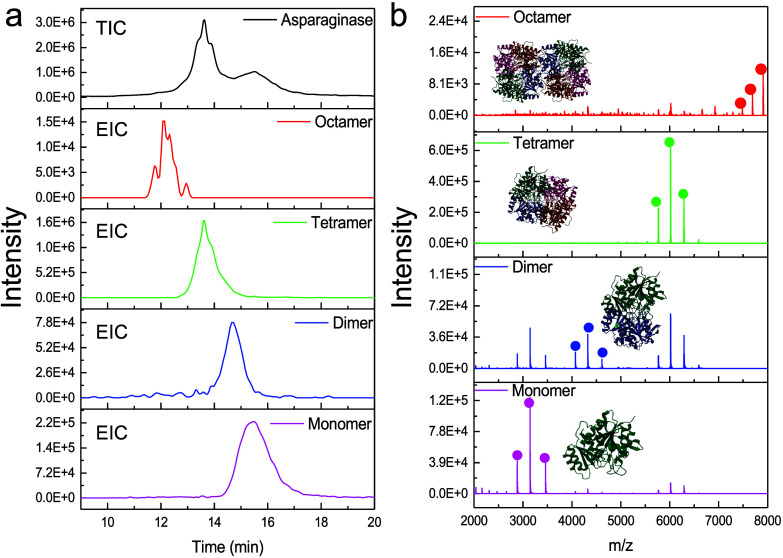
(a) TIC and EIC of l-asparaginase obtained by the nanoflow SEC-nMS method. (b) MS spectra of monomer, dimer, tetramer, and octamer of l-asparaginase. 50 nL of l-asparaginase with a concentration of 1 mg mL^–1^ was injected into the system and eluted with 200 mM AmAc. The isCID value is 15 eV. The *m*/*z* values used for EIC in (a) can be found in Table S5.

### Analysis of a Urine Sample from a Pregnant Donor

Finally, we applied our nanoSEC-nMS method to analyze a urine sample from a pregnant woman to showcase the capability of our method in preserving noncovalent protein complexes within complex biological matrices containing multiple proteins. A sample of urine collected from week 10 was analyzed, aiming to measure human chronic gonadotropin (hCG). hCG is a heterodimeric hormone of approximately 37 kDa, crucial in pregnancy and expressed in high concentrations, along with other hormones, in urine during pregnancy. It is composed of alpha and beta chains combined by noncovalent interactions.^54^ Measuring this protein by mass spectrometry presents significant analytical challenges due to its extensive (25–41% mass) and variable glycosylation (N- and O-linked glycans on both subunits, yielding numerous glycoforms).^55–59^

The analysis of hCG proteoform distribution could be highly interesting in clinical investigation as modification of this protein (e.g., hyperglycosylation) have been observed in diseases like cancer.^56^ However, to the best of our knowledge, no previous native MS study attempted to measure the hCG complex in urine samples. To approach this, we first concentrated the urine sample using spin filters (concentrated 10 times using 30 kDa MW cutoff). This resulted in a sample with high protein concentrations (1.4 mg mL^–1^, as measured by the Bradford assay). The concentrated urine sample and Ovitrelle, a recombinant hCG sample (also purified by spin filters), were reduced and analyzed by SDS-PAGE. As shown in Figure S13, five main protein bands with varying MWs (20–100 kDa) were observed in the urine sample, and three of these (MW 20–40 kDa) migrated to similar distances as Ovitrelle. We suggest that these may be the alpha and beta chains of hCG (UV area accounting for about 50% of the total protein intensity), and the difference in migration distance may be related to the different glycosylation patterns between the recombinantly expressed and endogenous forms. Interestingly high MW species around 80–100 kDa were also observed.

The nonreduced urine sample was analyzed using our nanoSEC-nMS method, injecting 50 nL corresponding to approximately 70 ng of proteins. This level of sensitivity would not have been possible with microflow SEC-MS (where about 1 μg of purified reference protein is typically injected). We observed an MS signal indicative of high mass species (2000 to 5000 *m*/*z*) eluting between 9 and 15 min. As shown in Figure S14, the SEC separation allowed to resolve higher MW and heterogeneous species (9 to 12 min), which could not be mass resolved from a group of smaller proteoforms of proteins that presented clear MS spectra (12 to 15 min, Figure 5a) and later lower molecular weight contaminants (15 to 18 min). A complex mass spectrum was observed between 12 and 15 min with masses between 2800 and 3400 *m*/*z* (Figure 5b). Decovolution of these *m*/*z* revealed diverse proteoforms in the range between 32 to 36 kDa. We suggest that these can be related to hCG with varying glycosylation degrees, site occupancies, and sodiated adducts (Figure 5c). In comparison, the biopharmaceutical Ovitrelle run with the same method showed a simpler mass profile with distinct peaks of proteoform species between 35.5 and 39 kDa (Figures 5d to 5f), also eluting in the area around 13 to 16 min, confirming the ability of our method to preserve the dimeric protein. The differences in elution time between the urine hCG and Ovitrelle presumably come from the different proteoforms that may be present in these two proteins (similarly, the SDS-PAGE results also showed only partial MW overlap). To further investigate the urinary protein composition, we also analyzed it using HILIC-MS under denaturing conditions, following methods previously described.^39,60^ Under HILIC-MS analysis, the heterodimer is not preserved. Analysis of Ovitrelle revealed several glycoforms, within which the alpha chain (approximately 14 kDa) and two types of beta chain (22 and 24 kDa) could be resolved and tentatively identified (Figure S15).^61^ Our HILIC-MS results agreed with previous subunit analysis and native MS data.^62^ HILIC-MS analysis of the urine sample (Figures S16 and S17) revealed two main proteins of approximately 13 kDa and 21 kDa, which can be representative of the alpha and beta chains of hCG, respectively.

**Figure 5:**
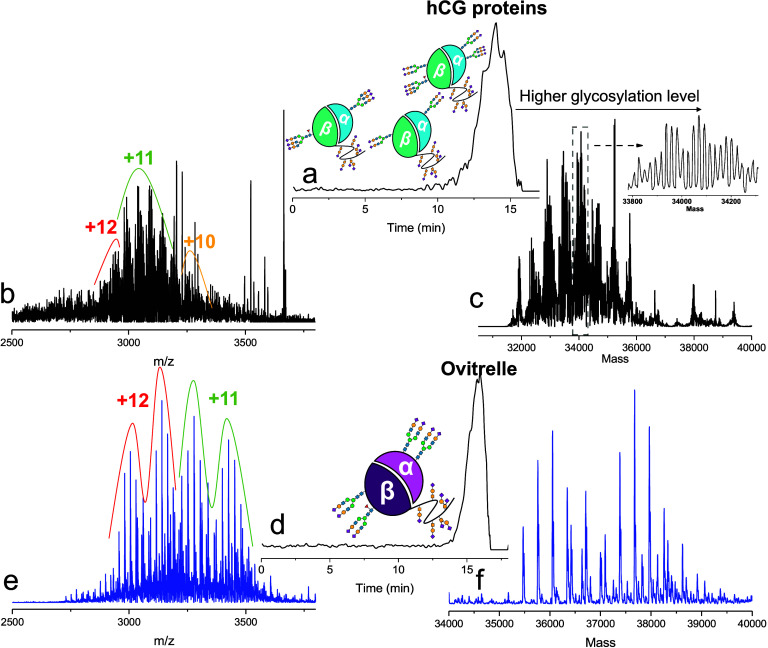
Analysis of urine samples and Ovitrelle with the nanoSEC-nMS: (a) EIC of the urine hCG samples; (b) MS spectrum of hCG proteins; (c) deconvoluted results of hCG proteins; (d) EIC of the Ovitrelle sample; (e) MS spectrum of Ovitrelle; (f) deconvoluted results of Ovitrelle. 50 nL of urine samples and Ovitrelle (0.5 mg mL^–1^) were injected into the system and eluted with 200 mM AmAc. The isCID values for both are 55 eV. Schematic illustration of the hCG structure and glycosylation is reported in (a) and (d). The *m*/*z* values (±0.5) used for EIC are 3042.72, 3205.08, 3522.91, 3664.44 (a) and 3442.57–3620.86 (d).

## Conclusion

Herein, we describe a nanoflow size-exclusion chromatography (SEC) – native MS (nMS) method to analyze proteins and protein complexes. In particular, we illustrate methods for packing capillary SEC columns, approaches to injecting samples, and emitters to achieve nanoESI-MS. The nanoflow SEC-nMS method provides conditions that enable the partial resolution of protein complexes in the liquid phase and their subsequent measurement by native mass spectrometry. Our method enables the analysis of proteins and complexes across a broad MW range (10 to 250 kDa) in their native states while preserving the noncovalently bound metal ions within the proteins. Additionally, only limited sample amounts are required (about 100 nL per injection, considering the loop of 50 nL and overfilling needed to ensure repeatable injections). Our method enables us to enhance the throughput of native MS, performing online desalting and oligomer separations within 25 min. Finally, we applied our method to analyze the Ovitrelle and urine pregnancy sample, proving the ability to preserve and sensitively detect noncovalent complexes in biological samples.

## Supplementary Material



## Notes

Further details on materials, sample preparation, and methods; column packing results; comparison of injection approaches; additional LC-MS results including the analysis of the urine from a pregnant donor by SDS-PAGE, nanoSEC-nMS and HILIC-MS; parameters used for MS charge deconvolution as well as equations and further details on the parameters used to obtain figures; CRediT author contribution (PDF)
