# Supplementary material for: Nanoflow Size Exclusion Chromatography–Native Mass Spectrometry of Intact Proteoforms and Protein Complexes
Source: Anal Chem. 2025 Jun 6;97(23):12241–50. doi: 10.1021/acs.analchem.5c01019 (PMC12177876; doi:10.1021/acs.analchem.5c01019)
Supplement: Supplementary file 1 [file ac5c01019_si_001.pdf]

Supplementary Information

Nanoflow Size Exclusion Chromatography – Native Mass Spectrometry of Intact Proteoforms and Protein Complexes

Ziran Zhai[†‡*], Thomas Holmark[†‡], Annika A. M. van der Zon[†‡], Vasilis Tseliou[†], Francesco G. Mutti[†], Alina Astefanei[†‡], and Andrea F.G. Gargano[†‡*]

[†]*Analytical Chemistry Group and Biocatalysis Group, Van't Hoff Institute for Molecular Sciences (HIMS), University of Amsterdam, Science Park 904, Amsterdam, 1098 XH, The Netherlands.*
[‡]*Centre for Analytical Sciences Amsterdam, Van't Hoff Institute for Molecular Sciences (HIMS), University of Amsterdam, Science Park 904, Amsterdam, 1098 XH, The Netherlands.*

*Corresponding author: Andrea F.G. Gargano; Ziran Zhai
E-mail: a.gargano@uva.nl
E-mail: z.zhai@uva.nl

*Corresponding author: Andrea F.G. Gargano; Ziran Zhai
E-mail: a.gargano@uva.nl, z.zhai@uva.nl

Table of Contents

S1. Experimental section S3
S1.1 Materials S3
S1.2 Performance evaluation of capillary trap columns. S3
S1.3 Trap-elute injection in the nanoSEC-nMS S4
S1.4 SDS-PAGE analysis of urine samples and Ovitrelle S4
S1.5 Bradford assay of urine samples S4
S1.6 Analysis of urine samples and Ovitrelle with HILIC-MS S5
S2. Supplemental Figures S6
S2.1 Preparation of capillary SEC columns S6
S2.2 Investigation of the coupling to nMS S7
S2.3 Comparison of injection approaches S8
S2.4 Additional nanoSEC-MS analysis of proteins and protein complexes S10
S2.5 SDS-PAGE, nanoSEC-nMS, HILIC-MS analysis of Ovitrelle and urine samples ... S13
S2.6 Investigation of mixed-bed trap columns S16
S3. Supplemental Tables S17
S4. Authors contributions: CRediT author statement S19
S5. References S19

S1. Experimental section

S1.1 Materials

Ammonium acetate (AmAc, ≥ 98%), sodium phosphate dibasic (≥ 98.5%), sodium phosphate monobasic (≥ 99.0%) %), trifluoroacetic acid (TFA, ≥ 99%), acrylamide (AA, 99%), N,N′-methylenebisacrylamide (MbA, 99%), 1-octanol (OctOH), dimethyl sulfoxide (DMSO > 99.9%), and 2,2′- azobisisobutyronitrile (AIBN, 98%) were purchased from Sigma-Aldrich (Steinheim, Germany). Methanol (MeOH, ULC-MS), 2-propanol (IPA, ULC-MS), and acetonitrile (ACN, ULC-MS) were purchased from Biosolve (Valkenswaard, The Netherlands). Ethylene glycol (>99.5%) and concanavalin A from Canavalia ensiformis are from Merck (Darmstadt, Germany). Tween 20, uracil (>99%), bovine serum albumin (BSA, ≥ 96%), myoglobin from equine skeletal muscle (Myo, ≥ 95%), ribonuclease A from bovine pancreas (RNase-A, ≥ 60%), ribonuclease B from bovine pancreas (RNase-B, ≥ 80%), alcohol dehydrogenase (ADH) from saccharomyces cerevisiae (≥ 300 units/mg), catalase from bovine liver (Cata, ≥ 20,000 units/mg), enolase from baker's yeast (≥ 50 units/mg), cytochrome c from equine heart (Cyt C, ≥ 95%), albumin from chicken egg white (Oval, ≥ 90%), γ-globulins from bovine blood (>95%), thyroglobulin from bovine thyroid (Thyro), lysozyme from chicken egg white (Lyso, ≥ 90%), and proteinase K from tritirachium album (PK, ≥ 30 units/mg) were purchased from Sigma-Aldrich (St. Louis, USA). Trastuzumab (Tra) was obtained from Roche (Grenzach-Wyhlen, Germany). The therapeutic enzyme L-asparaginase (ASNase, Paronal 10.000 I.U. equivalent to 40 mg powder per vial) produced in *E.coli* was obtained from Ghent University Hospital (Ghent Belgium). Ovitrelle (500 µg/mL hCG contained) is from Merck Europe B.V., Gustav Mahlerplein (SN: 45642318920, lot: BA099371). Ultrapure water (18.2 MΩcm) was produced by a Milli-Q purification system (Millipore, Bedford, MA, USA). Fused-silica capillaries (0.2 mm I.D., 0.36 mm O.D.) were purchased from CM Scientific (Silsden, UK). The frit kit (including formamide, Kasil-1, and Kasil-1624) was purchased from Next Advance (Troy, NY, USA). The cation exchange resins (Poly CAT A, PolySULFOETHYL A) and anion exchange resins (Poly WAX LP, Poly SAX LP) were obtained from PolyLC (Columbia, MD, USA). The column (1.0 × 300 mm × 4 µm × 250 Å) used for micro-flow SEC experiments was obtained from TOSOH (TSKgel SuperSW3000, Japan). The metal screen (part number: .5SR1-10) is from VICI (USA) and the plastic screen setup (part number: M-547 and M-133) is from IDEX Health & Science (USA).

S1.2 Performance evaluation of capillary trap columns.

The 0.5 mg mL^{-1} of acidic (Oval, BSA, Thyro, and γ-globulins) and basic proteins (Lyso, Cyt C, and RNase-B) were used to test the behaviors of mixed-bed trap columns by loading 1 µL of these proteins on the columns at 1.5 µL min^{-1} flow rate with the mobile phases of (A) 20 mM PBS (pH 7.0) and (B) 500 mM NaCl mixed with A (pH 7.0). To check the function of the mixed-bed trap and capillary SEC setup, proteins were first loaded on the trap column at 5 µL min^{-1} with 20 mM PBS (pH 7.0) for 2 min. Then the valve was switched, and the trapped samples were flushed into the capillary SEC column at 0.5 µL min^{-1} by the elution solvent of 200 mM PBS and 200 mM NaCl (pH 7.0). During the analysis, the monitored UV wavelengths were set at 214 nm, 256 nm, and 280 nm.

S1.3 Trap-elute injection in the nanoSEC-nMS

For trap injection mode, 5 μL of proteins with a concentration of 0.05 mg mL^{-1} in 20 mM AmAc solvents were loaded on the trap at a flow rate of 10 μL min^{-1} before the valve switched, and then they were flushed into the capillary SEC column by elution solvents of 200 or 400 mM AmAc. The internal diameters of the connecting tubes (fused silica capillaries) are below 50 μm.

To enhance the detection sensitivity and minimize the volume-induced peak-broadening, the mixed-bed (cation and anion) ion-exchange capillary trap columns were exploited to pre-concentrate the samples and narrow the peak widths. We prepared strong and weak ion-exchange trap columns using resins of various types: weak cation (Poly CAT), weak anion (Poly WAX LP), strong cation (PolySULFOETHYL A), and strong anion (Poly SAX LP), with pore sizes of 300 Å and 1000 Å. The packing solvents used were 50 mM PBS at pH 7.0 (low ionic strength), which allowed cation and anion resins to bind together uniformly, forming a relatively homogeneous column matrix. We tested the trapping capabilities of these columns using a range of acidic (ovalbumin, γ-globulins, BSA, thyroglobulin) and basic proteins (cytochrome C, lysozyme, and ribonuclease B). Both strong and weak ion-exchange trap columns can successfully capture these proteins, indicating their potential for handling a variety of complex samples. We then evaluated the effect of pore sizes on protein behaviors by comparing the obtained peak areas and widths. Figure S18 shows that while the larger pore size (1000 Å) produced slightly higher peak areas, the smaller pore size (300 Å) effectively reduced the band broadening. Consequently, we selected the strong ion exchange trap column with a 300 Å pore size for the next experiments. In addition, we found that the back-flush method outperformed the front-flush method in terms of peak areas and widths when eluting proteins from the trap columns (Figure S19).

S1.4 SDS-PAGE analysis of urine samples and Ovitrelle

SDS-PAGE analysis was conducted using a 12% (w/v) separating gel and a 4% (w/v) stacking gel. A 20 μL aliquot of the concentrated sample was mixed with 20 μL of Laemmli SDS sample buffer, which contained 4% of a 10% (w/v) SDS solution, 20% glycerol, 120 mM Tris-HCl (pH 6.8), 0.02% (w/v) bromophenol blue, and 2% (v/v) β-mercaptoethanol as a reducing agent. Similarly, 20 μL of the Ovitrelle (0.5 mg/mL) was combined with 20 μL of the same sample buffer. All samples were heated at 95 °C for 5 minutes. Electrophoresis was performed using a Mini-PROTEAN Tetra system (Bio-Rad) at a constant voltage of 160 V. Protein bands were visualized by Coomassie staining.

S1.5 Bradford assay of urine samples

The concentrations of urine samples were determined using the Bradford assay with bovine serum albumin (BSA) as the standard. A standard calibration curve was prepared by mixing 980 μL of Bradford reagent with 20 μL of BSA solutions at known concentrations ranging from 0.2 to 1.0 mg/mL, prepared by serial dilution from a 10 mg/mL BSA stock solution in 50 mM potassium phosphate buffer (pH 7.5). After thorough mixing and incubation for 5 minutes at room temperature, the absorbance at 595 nm was measured using a UV-Vis spectrophotometer. A linear calibration curve

was constructed by plotting absorbance against the known protein concentration. The urine samples were diluted appropriately to ensure their absorbance values fell within the linear range of the standard curve. The concentrations of the diluted samples were then calculated using the linear equation of the standard curve, and final protein concentrations were determined by correcting for the corresponding dilution factors.

S1.6 Analysis of urine samples and Ovitrelle with HILIC-MS

For HILIC-MS analysis, a similar setup reported by Zhai et al. was used.[1] Briefly, a trap column (5 mm × 0.3 mm I.D. × 5 µm, Thermo) with C4 stationary phase was used to inject samples. The urine samples and Ovitrelle (0.02 mg/mL) were first loaded on the trap column with 2% ACN at 10 µL/min using a loading pump. After 2 min, the valve was switched and the samples were eluted at 1 µL/min flow rate by the mobile phases consisting of 2% ACN containing 0.1% TFA (A) and 98% ACN containing 0.1% TFA (B) according to a gradient method (0 – 4 min, 94%B to 75%B; 4 – 5.875 min, 75%B to 60%B; 5.875 – 5.9 min, 60%B; 5.9 – 14.23 min, 60% to 45%; 14.23 – 16 min, 45%B to 20%B). The injection volume is 1 µL, and the temperature was set at 50 °C. The HILIC column (150 mm × 0.2 mm I.D.) was home-made with a polymerization mixture containing 13.75% AA, 11.25% MbA, 21.5% OctOH, 53.5% DMSO, and 1% AIBN, all by weight, according to the procedures described by Passamonti *et al.*[2] The MS parameters are as follows: spray voltage of 2.0 kV, transfer-capillary temperature of 275 °C, S-lens of RF 100, scan range: 600 to 4000 m/z, isCID of 15 eV, microscans of 10, resolution of 17,500 or 140,000; AGC target of 1 x 10^6; maximum IT of 200 ms.

S2. Supplemental Figures

S2.1 Preparation of capillary SEC columns

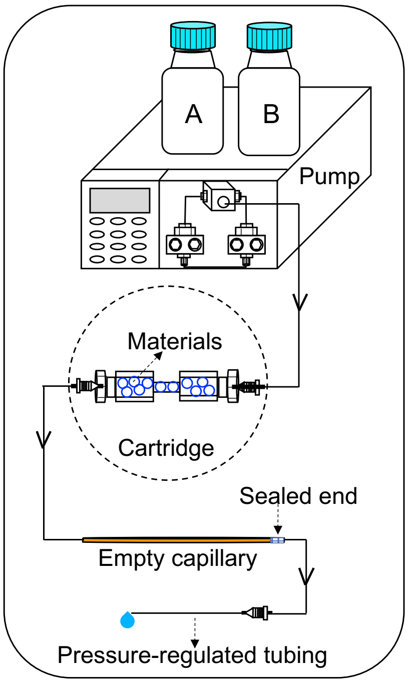

Figure S1. Schematic illustration of the packing station for the preparation of capillary columns.

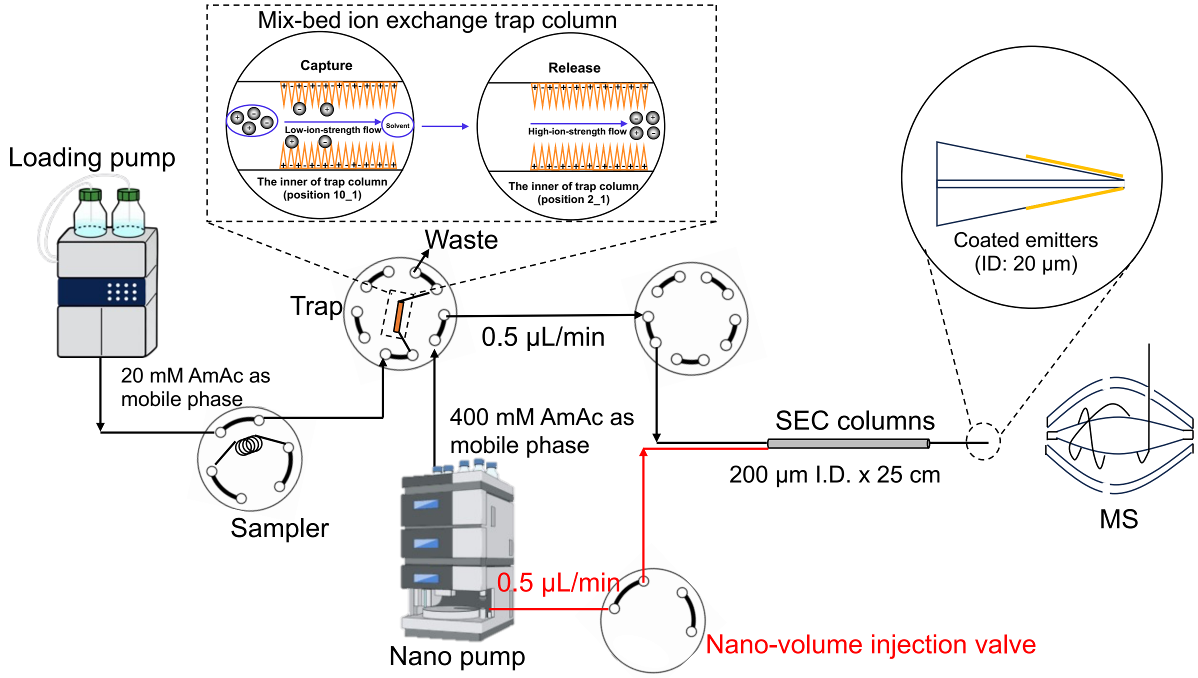

Figure S2. The workflow of nanoflow size exclusion chromatography–native MS system. The black line represents the trap injection mode; the red line represents the nano-volume injection mode.

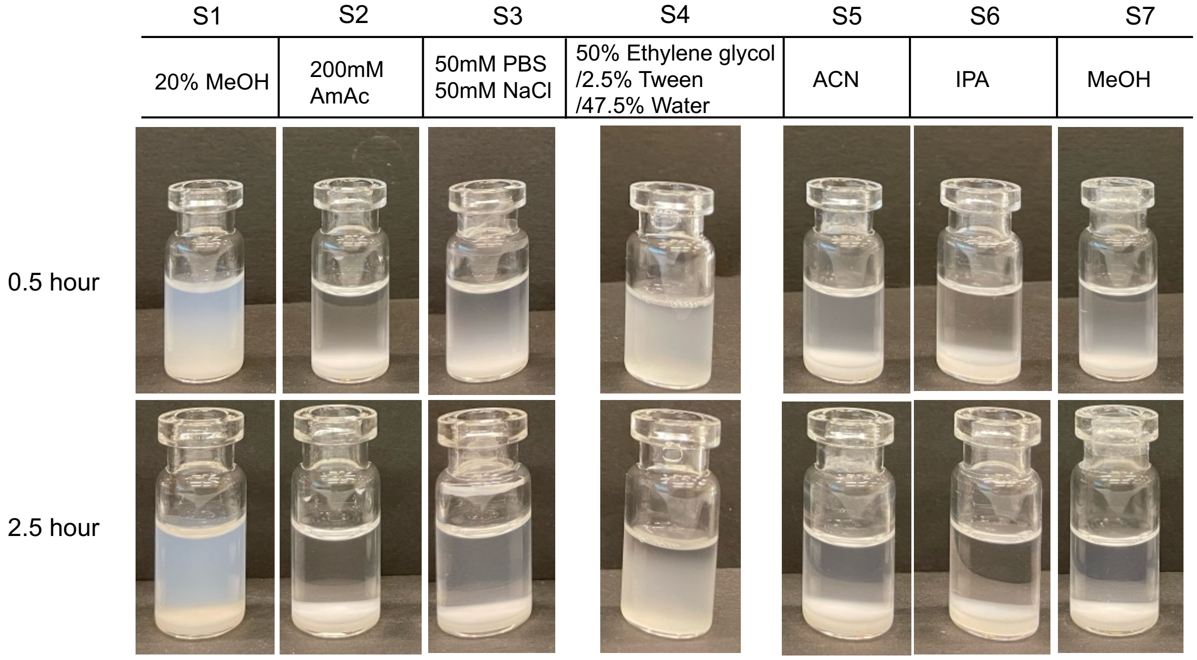

Figure S3. The performance test of SEC resins (250 Å × 5 μm) after being suspended in various solvents for 0.5 and 2.5 hours. The solvents are S1: 20% MeOH, S2: 200 mM AmAc, S3: 50 mM PBS/ 50 mM NaCl, S4: 50% ethylene glycol/2.5% Tween/47.5% water, S5: ACN, S6: IPA, and S7: MeOH.

S2.2 Investigation of the coupling to nMS

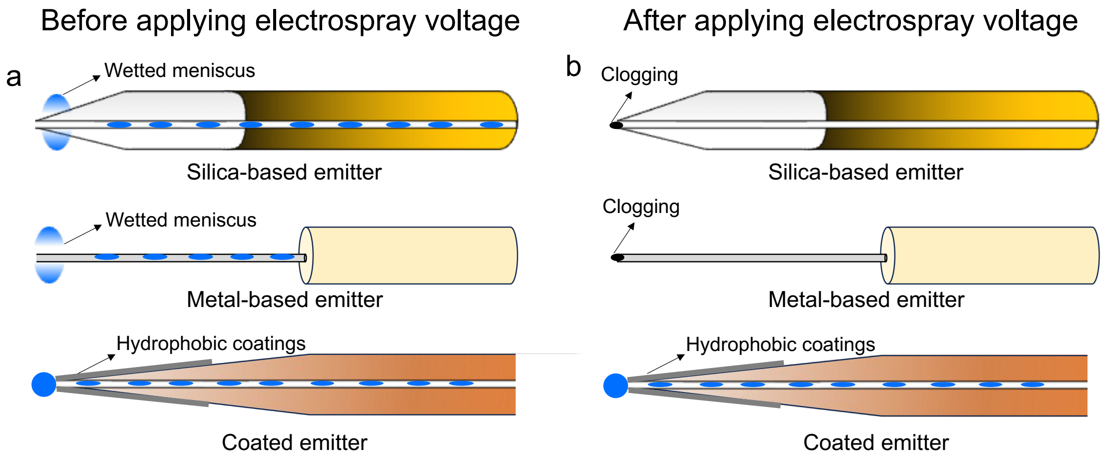

Figure S4. Schematic illustration of the performances of different emitters under a high concentration of volatile salts before (a) and after applying electrospray voltage (b).

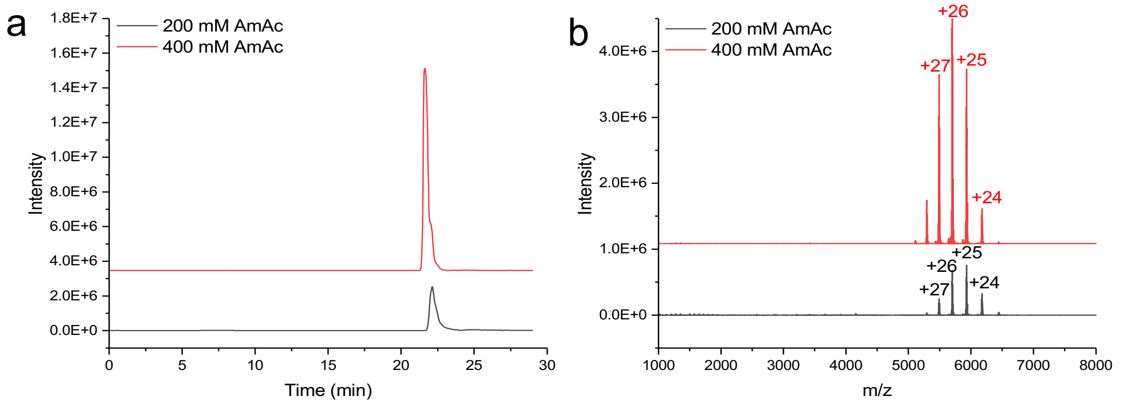

Figure S5. Comparison of different concentrations of mobile phase (200 mM AmAc and 400 mM AmAc) used to elute proteins from mixed-bed trap columns in the nanoflow SEC-nMS. (a) EIC of trastuzumab. (b) Mass spectra of trastuzumab. 5 μL of the proteins were injected with a concentration of 0.05 mg/mL. The value of isCID: 55 eV. The m/z values used to extract these peaks are: 5294.70, 5490.50, 5701.64, 5929.65, 6176.72, 6445.20 for 200 mM AmAc; 5294.70, 5490.50, 5701.64, 5929.65, 6176.72, 6445.20 for 400 mM AmAc.

S2.3 Comparison of injection approaches

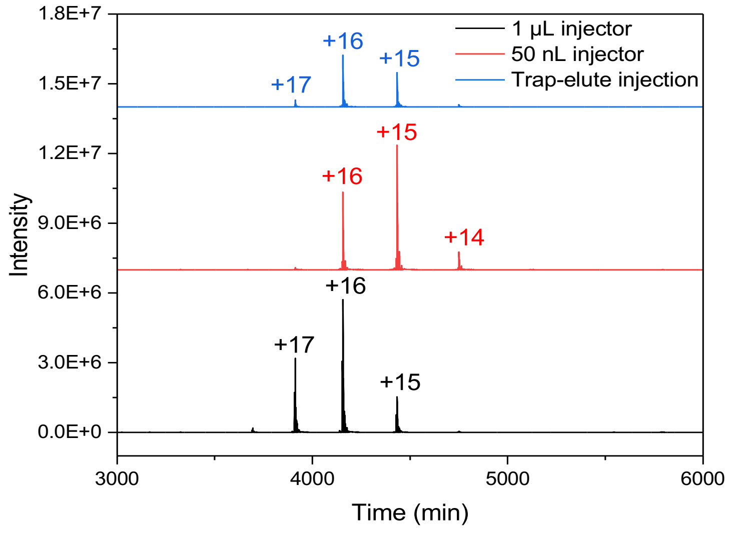

Figure S6. MS spectra of BSA measured by nanoflow SEC–nMS with different injection ways: 1μL injector, 50 nL injector, and trap-elute injection.

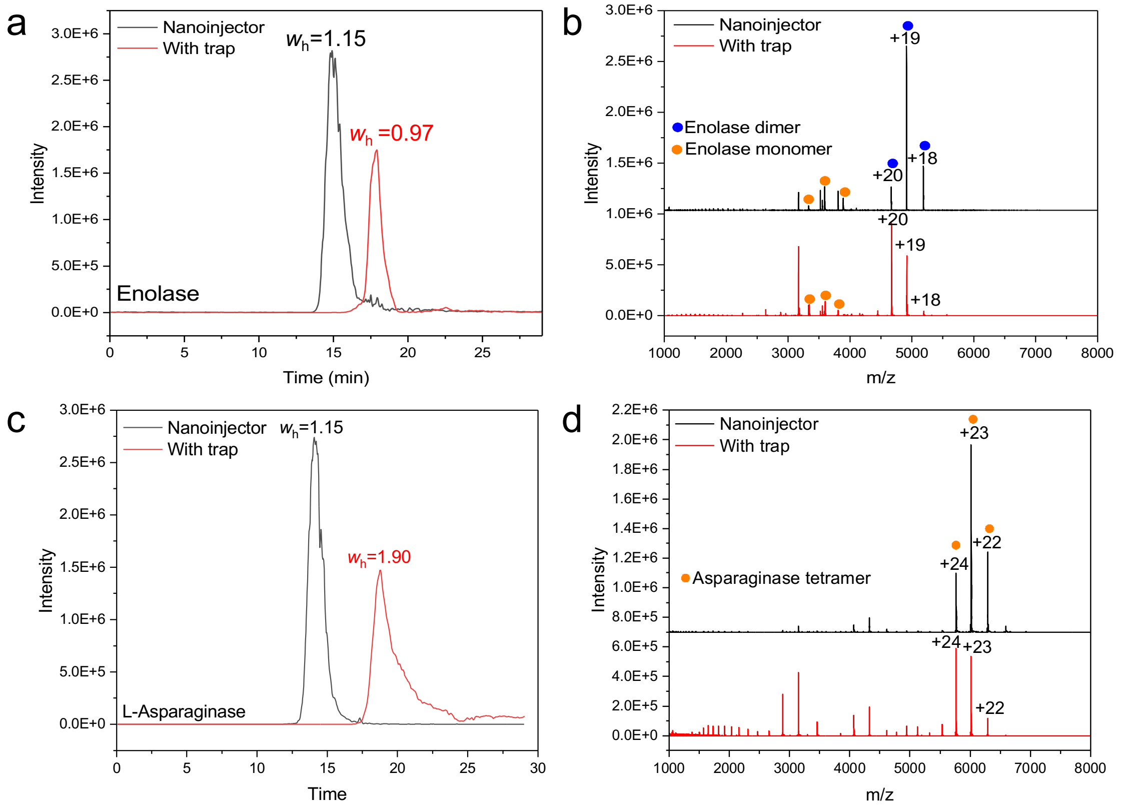

Figure S7. Comparison of the injection approaches between nanoinjector and the trap column in nanoflow SEC-nMS method. (a, c) EIC of enolase and L-asparaginase. (b, d) Mass spectra of enolase and L-asparaginase. With the nanoinjector, 50 nL of proteins at the concentration of 1 mg mL^{-1} were injected into the system. With the trap column: 5 μL of proteins at the concentration of 0.01 mg mL^{-1} were injected into the system. The elution solvent is 200 mM AmAc. The isCID values are 55 and 15 eV.

S2.4 Additional nanoSEC-MS analysis of proteins and protein complexes

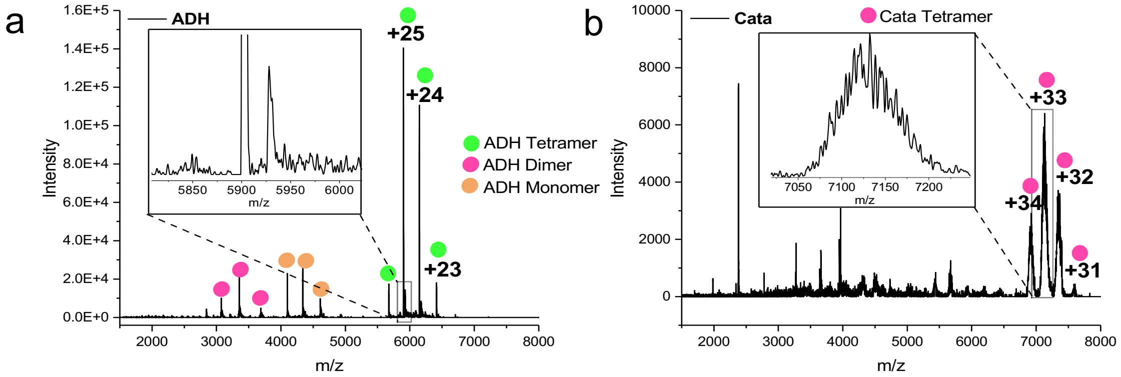

Figure S8. Mass spectra of various proteins and complexes measured by the nanoflow SEC-nMS. (a) Alcohol dehydrogenase (ADH). (b) Catalase (Cata). 50 nL of these proteins (1 mg mL^{-1}) were injected into the system and eluted with 200 mM AmAc. Values of isCID for these proteins are 55 and 0 eV respectively.

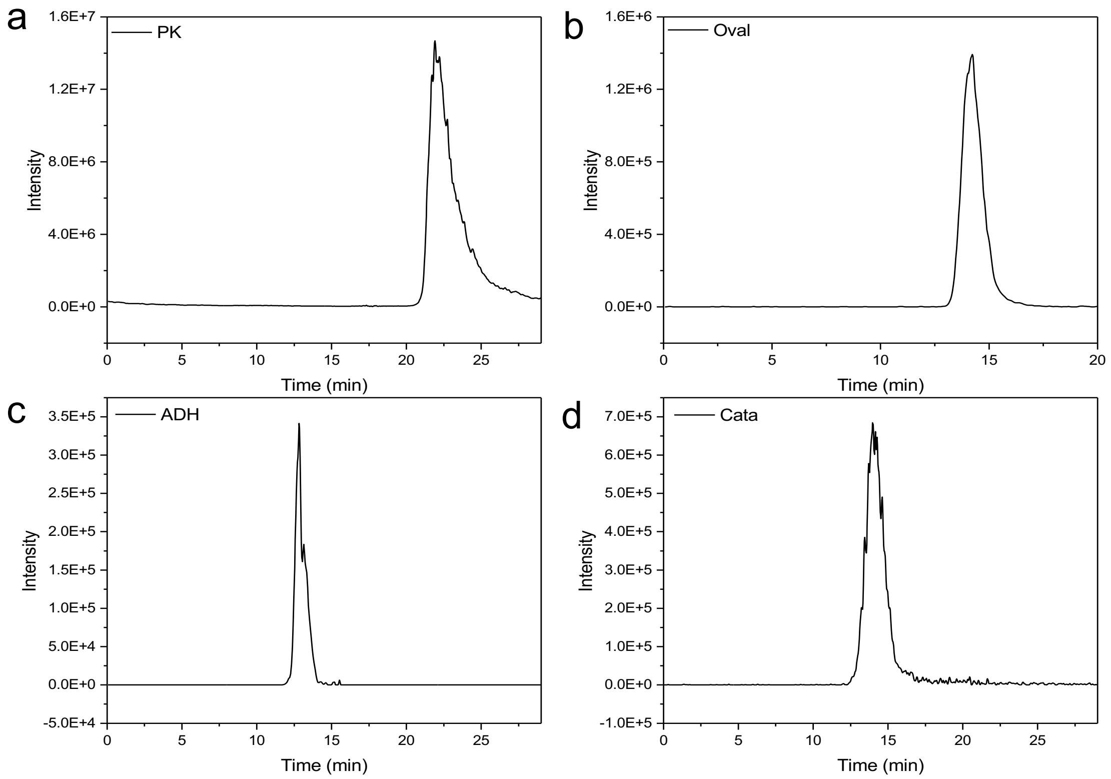

Figure S9. EIC of various proteins measured by the nanoflow SEC-nMS. (a) Proteinase K (PK). (b) Ovalbumin (Oval). (c) Alcohol dehydrogenase (ADH). (d) Catalase (Cata). The m/z values used to extract these peaks are: 2893.96, 3215.40, 3617.21 for PK; 3398.45, 3695.11, 4030.94, 4444.91 for Oval; 6871.76-7418.47 for Cata; 5674.53, 5901.35, 6147.14, 6414.45 for ADH.

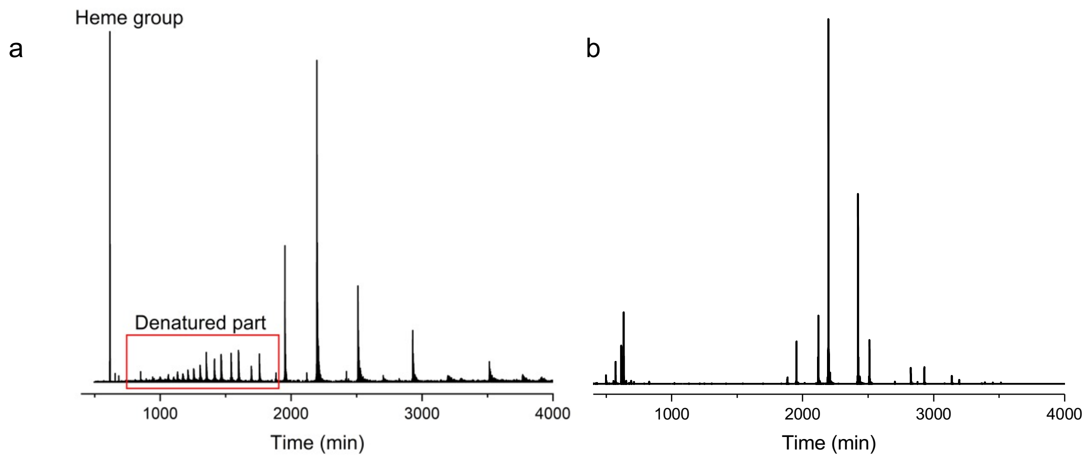

Figure S10. Mass spectrum of myoglobin measured by microflow SEC-MS (a) and nanoSEC-MS (b). The heme group was detached and some denatured parts were generated during the analysis in (a) compared with (b).

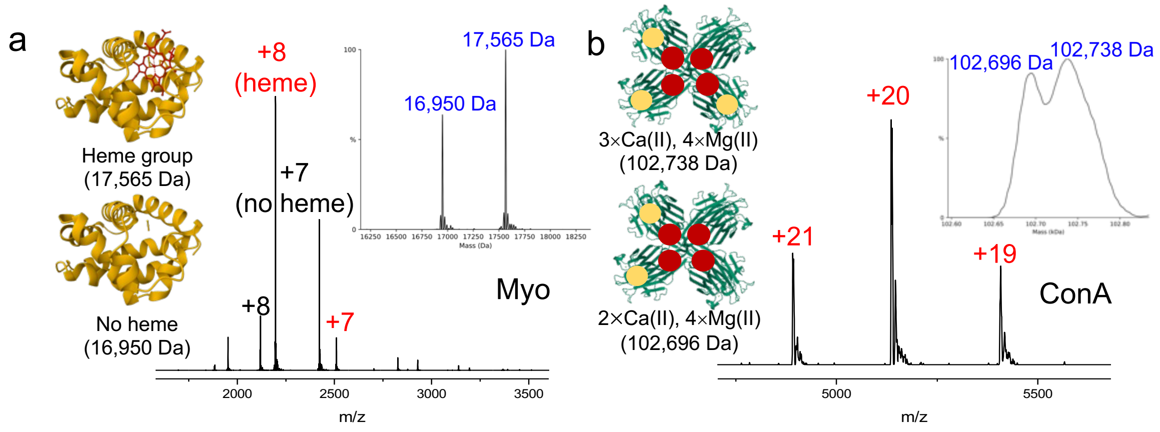

Figure S11. Analysis of proteins coordinated with ions by nanoflow SEC-nMS. (a) Myoglobin (Myo); (b) Concanavalin A (ConA). The 50 nL of proteins (1 mg mL^{-1}) were injected into the system and eluted with 200 mM AmAc. The isCID values are 55 eV (Myo) and 15 (ConA). The mass of each protein was obtained with the deconvolution methods shown in Table S1.

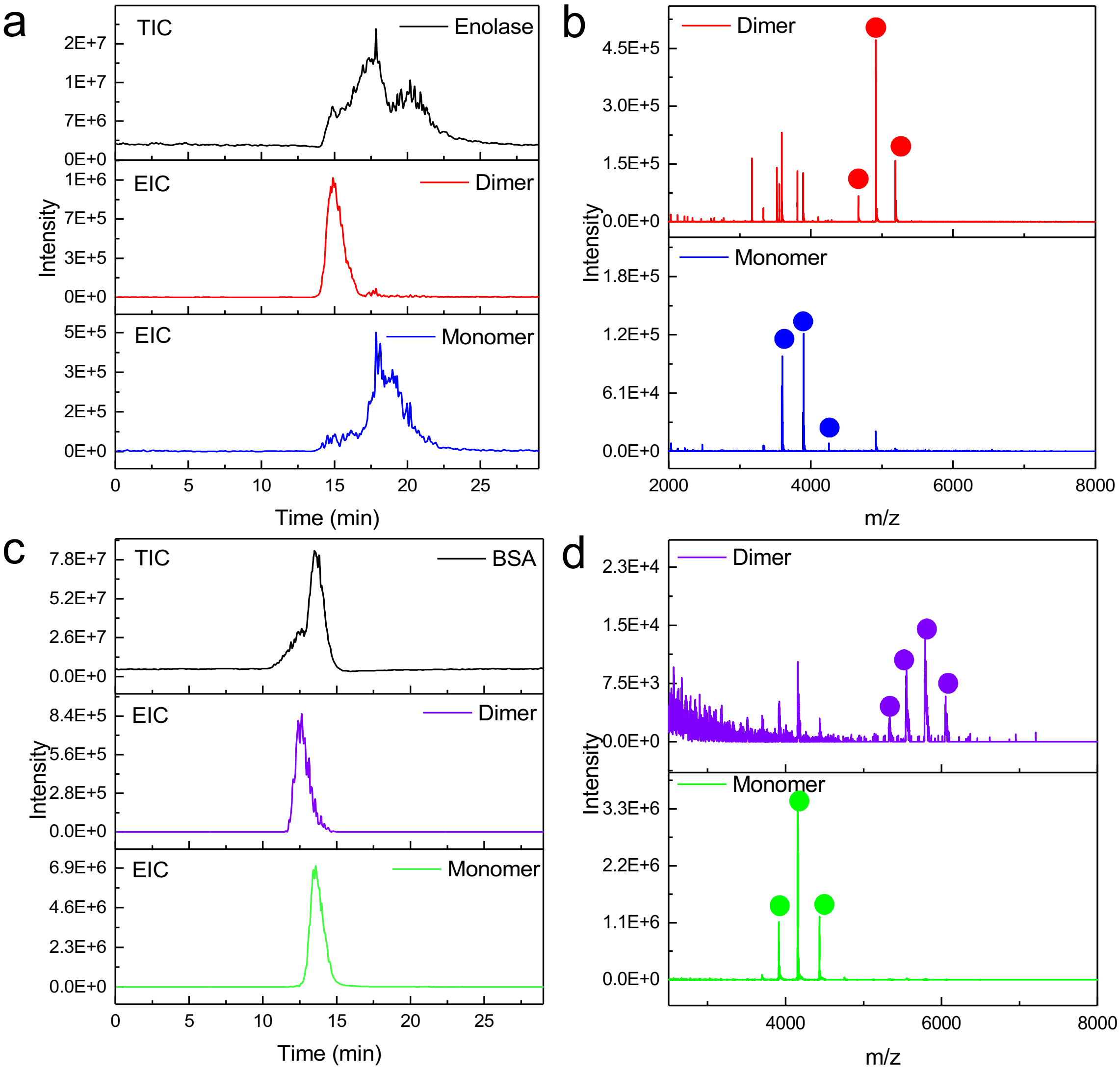

Figure S12. TIC and EIC of Enolase (a) and BSA (c) were obtained by nanoflow SEC–nMS method. (b) MS spectra of various oligomeric forms of Enolase (b) and BSA (d). The m/z values used to extract these peaks are: 4668.41, 4914.06, 5187.04 for the dimer of enolase; 3599.56, 3899.44, 4253.84 for the monomer of enolase; 3912.15, 4156.64, 4433.63 for the monomer of BSA; 5548.51, 5764.59-5830.58 for the dimer of BSA. The isCID values for enolase and BSA are 15 eV and 55 eV.

S2.5 SDS-PAGE, nanoSEC-nMS, HILIC-MS analysis of Ovitrelle and urine samples

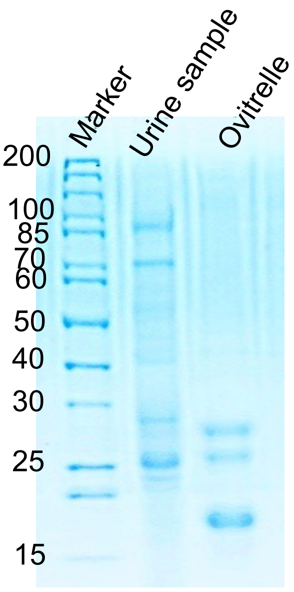

Figure S13. SDS-PAGE results of urine samples and Ovitrelle.

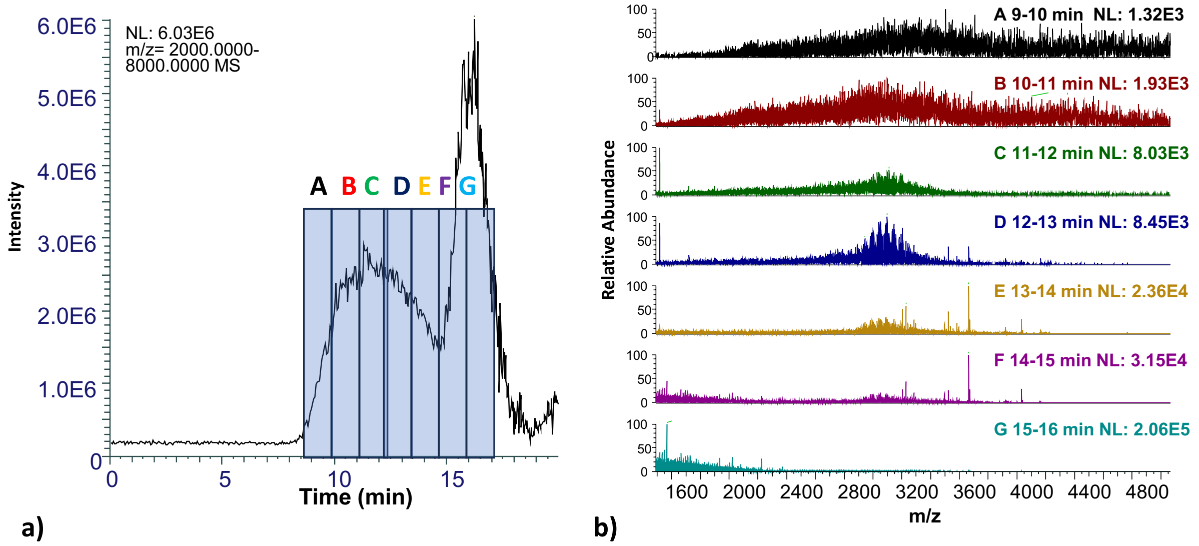

Figure S14. a) TIC (mass range 2000-8000) of the urine samples analyzed by nanoSEC-nMS. b) Average mass spectra of 1 min between 9 and 16 min, showing the respective MS observed.

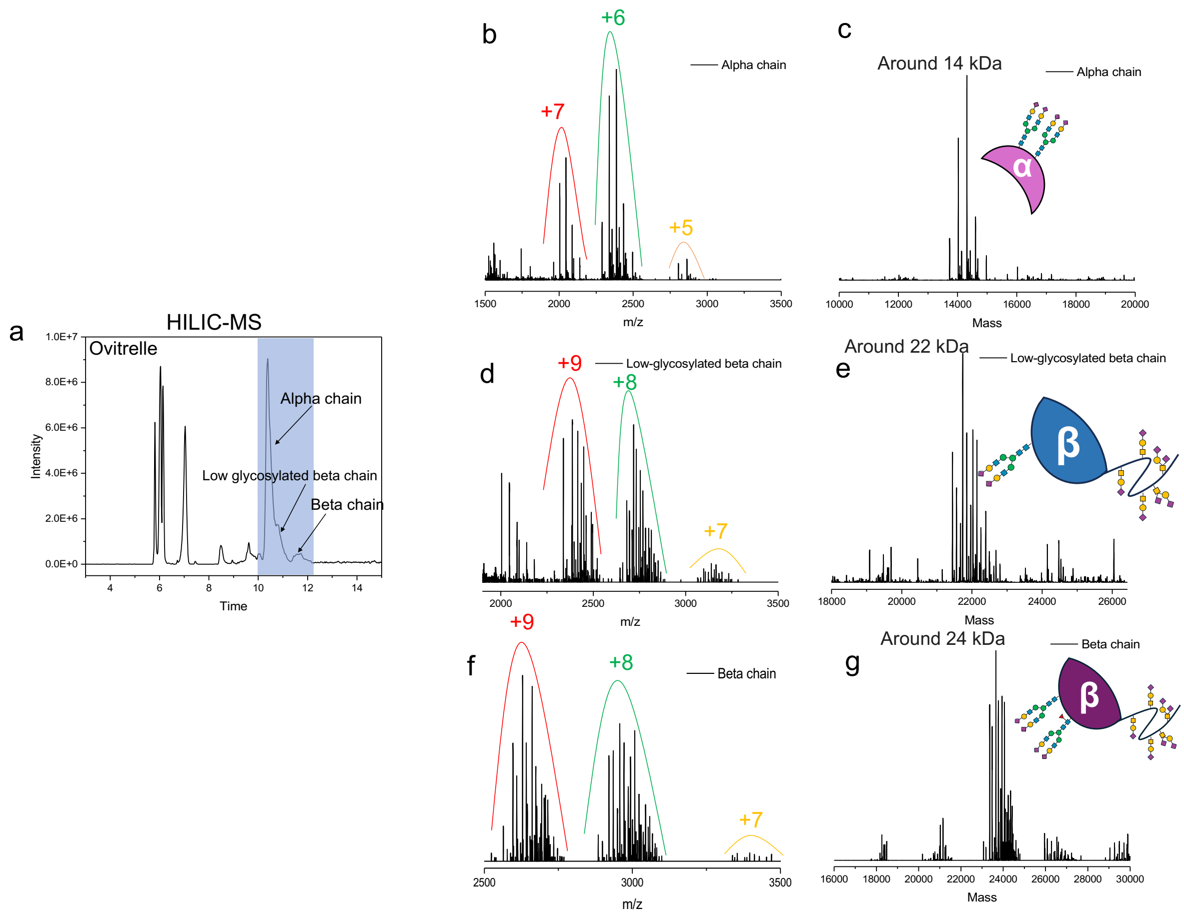

Figure S15. Analysis of Ovitrelle with the HILIC-MS. (a) TIC of Ovitrelle with the mass range from 2000 to 4000 m/z including tentative assignment based on intact mass. (b, d, f) Mass spectra of alpha chain, low-glycosylated beta chain, and beta chain.; (c, e, g) Deconvolution results of alpha chain, low-glycosylated beta chain, and beta chain.

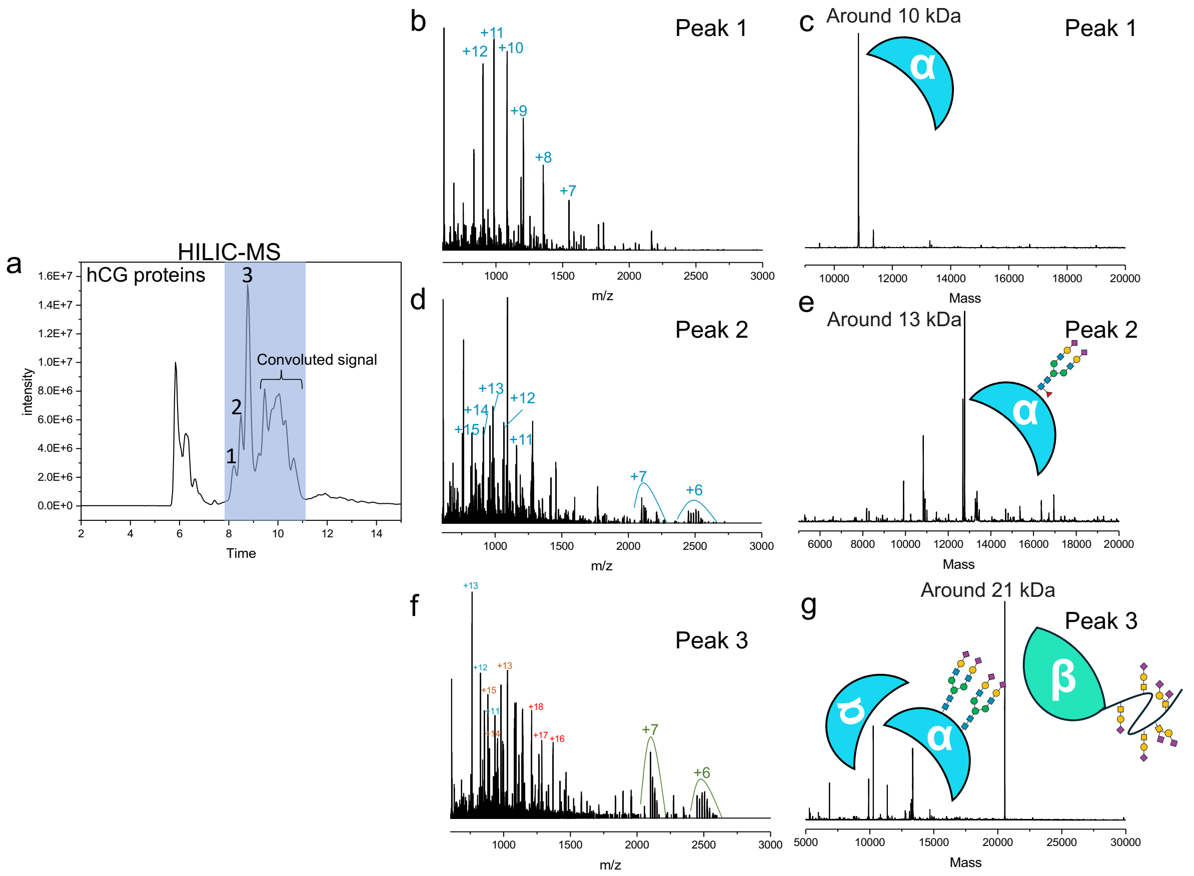

Figure S16. Analysis of urine samples with the HILIC-MS. (a) TIC (high resolution 140 k) of urine samples with the mass range from 2000 to 4000 m/z. (b, d, f) Mass spectra of peak 1 to peak 3. (c, e, g) Deconvolution results of peak 1 to peak 3. Peak 1 represents the alpha chain, peak 2 represents the glycosylated alpha chain, and peak 3 includes alpha chain, glycosylated alpha chain, and beta chain.

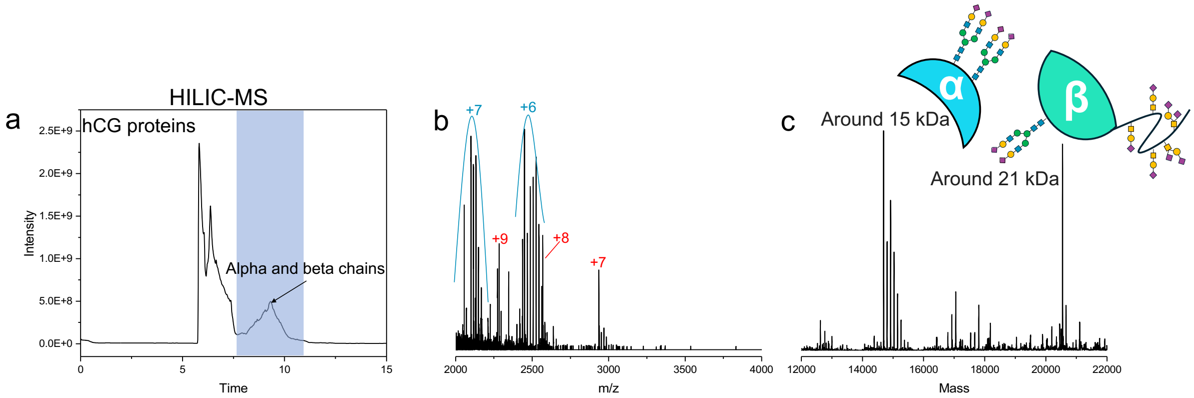

Figure S17. Analysis of urine samples with the HILIC-MS. (a) TIC (low resolution 17.5 k) of urine samples. (b) Mass spectrum of hCG proteins; (c) Deconvolution results of hCG proteins. The alpha chain (15 kDa) and beta chain (21 kDa) can be clearly observed.

S2.6 Investigation of mixed-bed trap columns

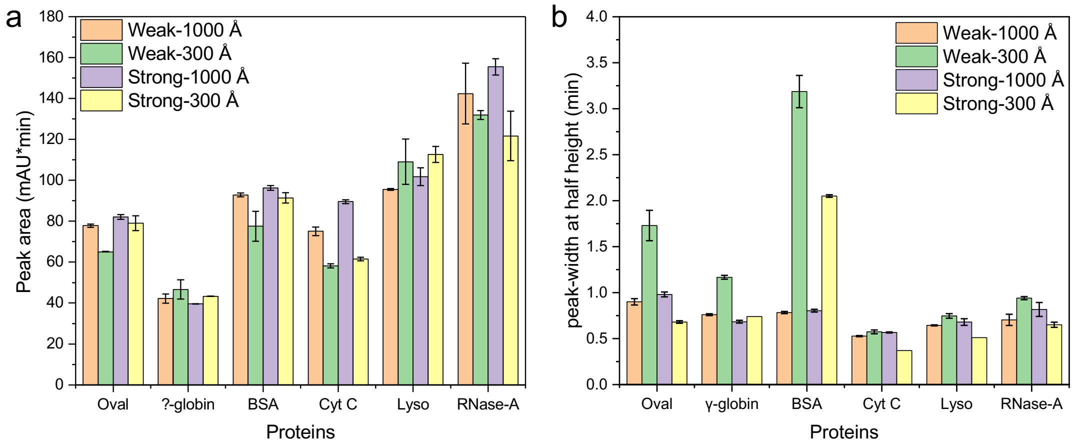

Figure S18. Peak areas (a) and peak-width at half height (b) of various proteins measured by strong and weak mixed-bed ion-exchange trap columns with different pore sizes (1000 Å and 300 Å).

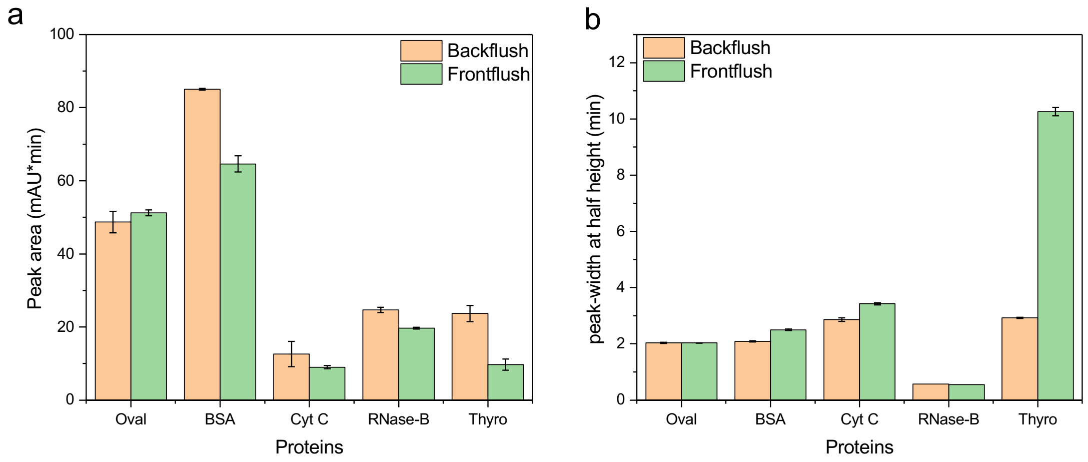

Figure S19. Comparison of the effects of backflush and front-flush on the elution of proteins with mixed-bed ion-exchange trap columns in terms of peak area (a) and peak-width at half height (b).

S3. Supplemental Tables

Table S1. Parameters used for the deconvolution with UniDec.

Parameters	Values
m/z picking areas	Depending on proteins
Charge range	1 to 50
Mass range	Around MW values
Sample mass every	1 Da
Peak detection range	1 Da
Peak detection threshold	0.01
Substract curved	0 or 10 (hCG)
Charge smooth width	1 or -1 (hCG)
Mass smooth width	0 or -2 (hCG)
Peak width	0.85 or 0.5 (hCG)
Mass difference	0 or 22 (hCG)
Smooth nearby points	Some

Table S2. The list of formulas.

Name	Formula	Symbol explanation
F1	Volume load (%) = $\frac{V_I}{0.6 * V_c}$ * 100%	V_I: injection volume; V_c: column volume
F2	Elution volume = $W * F$	W: peak width; F: flowrate
F3	Theory plates (N) = 5.54 * ($\frac{T_R}{w_h}$) ^2	T_R: retention time; w_h: peak-width at half height
F4	Asymmetry (A) = $\frac{RW_{5\%}+LW_{5\%}}{2*LW_{5\%}}$	$RW_{5\%}$: right peak width in 5% of the peak height; $LW_{5\%}$: left peak width in 5% of the peak height
F5	SEC column volume (V) $= p * L * \pi r^2$	p: porosity (60%); L: column length; r: the radius of capillary columns
F6	Linear flow velocity (L) $= p * \frac{V}{6\pi r^2}$	L: linear flow velocity (mm/s); p: porosity (60%); V: volumetric flow rate (mL/min); r: the radius of capillary columns (cm).

Table S3. Lists of peak asymmetry values for seven packing solvents in Figure 1b.

Packing solvents	Peak asymmetry
S1: 20% MeOH	1.06
S2: 200 mM AmAc	1.05
S3: 50 mM PBS/50 mM NaCl	1.05
S4: 50% ethylene glycol/2.5% Tween/47.5% water	0.87
S5: ACN	1.49
S6: IPA	1.47
S7: MeOH	1.55

Table S4. Recovery of using the trap-elute injection method.

Proteins	Areas (nanoinjector)	Areas (with trap)	Recovery (%)	Ave.Recovery (%)
BSA	483537903	284006169	58.74	59.18%
Enolase	225509211	113627785	50.39	
Asparaginase	206562090	141329764	68.42	

Note: the data of BSA, enolase, and asparaginase are used from Figure 2b, Figure S7.

Table S5. Lists of m/z values for EIC in Figures 2a, 2b, and 5a.

Peak	m/z values for EIC
Microflow	3691.51, 3908.58, 4152.82, 4429.58
Nanoflow	3694.84, 3912.19, 4156.63, 4433.71
Trap-elute injection	3912.14, 4156.60, 4433.63
1 µL injector	3912.19, 4156.63, 4433.71
50 nL injector	4156.61, 4433.63, 4750.29
Asparaginase_Octamer	7483.53, 7688.94, 7908.12
Asparaginase_Tetramer	5766.59, 6017.23, 6290.74
Asparaginase_Dimer	4071.45, 4324.95, 4613.15
Asparaginase_Monomer	2883.59, 3145.68, 3460.11

Table S6. Theoretical and observed average masses for the protein standards measured with nanoSEC-nMS.

Proteins	UniProt ID	Theoretical MW	Observed MW	Mass Error (Da)	Mass Error (ppm)
Myoglobin (heme group)	P68082	17,566	17,565	1	56.9
Myoglobin (no heme)		16,951	16,950	1	58.9
Enolase (4×Mg(II)-unbound)	P00924	93,342[3]	93,348	-6	64.3
Enolase (4×Mg(II)-bound)		93,438	93,443	-5	53.1
ConA (4×Mn(II), 2×Ca(II))	P02866	102692	102696	-4	39.0
ConA (4×Mn(II), 3×Ca(II))		102732	102738	-6	58.4
ADH (2×Zn(II)-unbound)	P00330	147,515[4] (+Na^+)	147,516	-1	6.8
ADH (2×Zn(II)-bound)		147,643[4] (+Na^+)	147,642	1	6.8

S4. Authors contributions: CRediT author statement

Ziran Zhai: Conceptualization, Methodology, Investigation, Writing - Original Draft, Writing - Review & Editing. **Thomas Holmark, Annika A. M. van der Zon, Vasilis Tseliou, and Francesco G. Mutti**: Investigation. **Alina Astefanei**: Resources**. Andrea F.G. Gargano**: Conceptualization, Methodology, Investigation, Project administration, Supervision, Resources, Writing - Original Draft, Writing - Review & Editing.

S5. References

(1) Zhai, Z.; Schoenmakers, P. J.; Gargano, A. F. G. Identification of heavily glycated proteoforms by hydrophilic-interaction liquid chromatography and native size-exclusion chromatography - High-resolution mass spectrometry. *Anal. Chim. Acta* **2024**, *1304*, 342543.
(2) Passamonti, M.; de Roos, C.; Schoenmakers, P. J.; Gargano, A. F. G. Poly(acrylamide-co-N,N'-methylenebisacrylamide) Monoliths for High-Peak-Capacity Hydrophilic-Interaction Chromatography-High-Resolution Mass Spectrometry of Intact Proteins at Low Trifluoroacetic Acid Content. *Anal. Chem.* **2021**, *93* (48), 16000-16007.
(3) Fischer, M. S.; Rogers, H. T.; Chapman, E. A.; Chan, H. J.; Krichel, B.; Gao, Z.; Larson, E. J.; Ge, Y. Online Mixed-Bed Ion Exchange Chromatography for Native Top-Down Proteomics of Complex Mixtures. *J. Proteome Res.* **2024**, *23* (7), 2315-2322.
(4) Schachner, L. F.; Ives, A. N.; McGee, J. P.; Melani, R. D.; Kafader, J. O.; Compton, P. D.; Patrie, S. M.; Kelleher, N. L. Standard Proteoforms and Their Complexes for Native Mass Spectrometry. *J. Am. Soc. Mass Spectrom.* **2019**, *30* (7), 1190-1198.
